# Interplay of Intrinsic and Synaptic Conductances in the Generation of High-Frequency Oscillations in Interneuronal Networks with Irregular Spiking

**DOI:** 10.1371/journal.pcbi.1003574

**Published:** 2014-05-01

**Authors:** Fabiano Baroni, Anthony N. Burkitt, David B. Grayden

**Affiliations:** 1 NeuroEngineering Laboratory, Dept. of Electrical & Electronic Engineering, University of Melbourne, Parkville, Victoria, Australia; 2 Centre for Neural Engineering, University of Melbourne, Parkville, Victoria, Australia; 3 Bionics Institute, East Melbourne, Victoria, Australia; Indiana University, United States of America

## Abstract

High-frequency oscillations (above 30 Hz) have been observed in sensory and higher-order brain areas, and are believed to constitute a general hallmark of functional neuronal activation. Fast inhibition in interneuronal networks has been suggested as a general mechanism for the generation of high-frequency oscillations. Certain classes of interneurons exhibit subthreshold oscillations, but the effect of this intrinsic neuronal property on the population rhythm is not completely understood. We study the influence of intrinsic damped subthreshold oscillations in the emergence of collective high-frequency oscillations, and elucidate the dynamical mechanisms that underlie this phenomenon. We simulate neuronal networks composed of either Integrate-and-Fire (IF) or Generalized Integrate-and-Fire (GIF) neurons. The IF model displays purely passive subthreshold dynamics, while the GIF model exhibits subthreshold damped oscillations. Individual neurons receive inhibitory synaptic currents mediated by spiking activity in their neighbors as well as noisy synaptic bombardment, and fire irregularly at a lower rate than population frequency. We identify three factors that affect the influence of single-neuron properties on synchronization mediated by inhibition: *i*) the firing rate response to the noisy background input, *ii*) the membrane potential distribution, and *iii*) the shape of Inhibitory Post-Synaptic Potentials (IPSPs). For hyperpolarizing inhibition, the GIF IPSP profile (factor *iii*)) exhibits post-inhibitory rebound, which induces a coherent spike-mediated depolarization across cells that greatly facilitates synchronous oscillations. This effect dominates the network dynamics, hence GIF networks display stronger oscillations than IF networks. However, the restorative current in the GIF neuron lowers firing rates and narrows the membrane potential distribution (factors *i*) and *ii*), respectively), which tend to decrease synchrony. If inhibition is shunting instead of hyperpolarizing, post-inhibitory rebound is not elicited and factors *i*) and *ii*) dominate, yielding lower synchrony in GIF networks than in IF networks.

## Author Summary

Neurons in the brain engage in collective oscillations at different frequencies. Gamma and high-gamma oscillations (30–100 Hz and higher) have been associated with cognitive functions, and are altered in psychiatric disorders such as schizophrenia and autism. Our understanding of how high-frequency oscillations are orchestrated in the brain is still limited, but it is necessary for the development of effective clinical approaches to the treatment of these disorders. Some neuron types exhibit dynamical properties that can favour synchronization. The theory of weakly coupled oscillators showed how the phase response of individual neurons can predict the patterns of phase relationships that are observed at the network level. However, neurons *in vivo* do not behave like regular oscillators, but fire irregularly in a regime dominated by fluctuations. Hence, which intrinsic dynamical properties matter for synchronization, and in which regime, is still an open question. Here, we show how single-cell damped subthreshold oscillations enhance synchrony in interneuronal networks by introducing a depolarizing component, mediated by post-inhibitory rebound, that is correlated among neurons due to common inhibitory input.

## Introduction

Fast oscillations (30–100 Hz and higher) have been observed in several brain areas, and have been proposed as a general substrate of neural computation [1]–[4]. Several decades of intense investigations, using both experimental [5], [6] and theoretical [7]–[13] approaches, have provided a detailed picture of how high-frequency oscillations are generated and modulated in the brain. Nevertheless, how intrinsic, single-cell dynamical properties affect high-frequency oscillations, and through which mechanisms, is only partially understood. In particular, most theoretical studies have focused on the mechanisms of collective synchronization in the regime where individual neurons fire regularly and can be considered as quasi-periodic oscillators [14]–[17]. However, cortical neurons *in vivo* generally exhibit highly variable spiking activity [18]. As we show in this study, the intrinsic neuronal properties that are more important for the generation of collective oscillations depend critically on the dynamical regime where individual neurons operate.

Experimental and theoretical work demonstrated a key role for inhibitory interneurons in the generation of high-frequency oscillations [19]. In particular, application of metabotropic glutamate agonists *in vitro* in appropriate doses can elicit gamma oscillations which are robust to pharmacological suppression of fast glutamate-dependent excitation, but not of fast 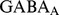 inhibition [6] (note, however, that some level of phasic excitation is generally required for gamma rhythmicity elicited by cholinergic or kainate agonists; see for example [20], [21]). Even more direct evidence comes from optogenetic experiments, where selective activation of fast-spiking interneurons has been shown to enhance gamma oscillations *in vivo* [22], [23].

Interneurons in the cortex and hippocampus are present in several subtypes, characterized by specific molecular, electrophysiological and dynamical properties, as well as postsynaptic cellular and subcellular targets [24], [25]. In particular, parvalbumin-positive fast-spiking basket cells have been shown to be causally related to the emergence of gamma oscillations [22]. These neurons are endowed with specific synaptic and intrinsic mechanisms that make them especially suitable for eliciting the temporally precise trains of Inhibitory Post-Synaptic Potentials (IPSPs) that are required for the generation of gamma oscillations [26]. Remarkably, they often exhibit intrinsic subthreshold oscillations or resonance in the gamma range, which have been attributed to the interplay between a persistent sodium conductance and a delayed-rectifier potassium conductance [27].

Intrinsic, single-cell oscillations have been observed in a variety of cell types [28], [29], including several types of inhibitory interneurons [27], [30]–[34]. Subthreshold oscillations have been proposed as a dynamical substrate for several computations at the single neuron level, including band-pass filtering [35], [36], recognition of temporally precise sequences of inputs [37], [38] and differential regulation of incoming connection strengths through spike timing-dependent plasticity rules [39].

Like pacemaker neurons in central pattern generators, it has been suggested that neurons with subthreshold oscillations play a key role in rhythmogenesis [40]. Being endowed with an intrinsic rhythm, just a few of these neurons can entrain a population of (mostly) passive cells to oscillate coherently through synaptic and/or ephaptic coupling. However, it has long been known that network oscillations can be produced even if individual neurons lack any oscillatory property, as a result of chemical and/or electrical synaptic interactions [41], [42].

Previous work has assessed the influence of single-cell intrinsic dynamics on network activity in the regular firing regime, where each neuron fires repetitively with little variation across cycles [14]–[17]. In this regime, Phase Response Curve (PRC) theory provides a suitable framework for the prediction of network activity from the dynamical characteristics of constituent neurons [43], [44]. The PRC of a regularly spiking neuron quantifies the phase shift that results from an infinitesimal perturbation as a function of the phase of the cycle at which the perturbation is applied. The shape of the PRC depends on the geometry of the limit cycle corresponding to tonic, regular spiking. However, cortical neurons *in vivo* generally exhibit highly variable spiking activity [18]. They dwell most of the time in the subthreshold regime and are driven beyond threshold by random fluctuations in their inputs. This variable activity at the single-cell level can nevertheless result in coherent, regular oscillations at the collective level [9], [10]. Features of the collective oscillations can be quantitatively predicted from the phase response of the neuronal and synaptic dynamics in the case of sinusoidal oscillations [9], [10] and also in the case of fully-developed, non-linear oscillations in networks of IF neurons driven by heterogeneous levels of DC currents [45].

The dynamical mechanisms by which intrinsic oscillations at the single-cell level affect global network oscillations are very different depending on the dynamical regime in which individual neurons operate. If individual neurons fire in each cycle of the collective oscillation (i.e., in the mean-driven regime), the geometry of the single-cell periodic attractor corresponding to the regular spiking regime enables one to predict and understand the population rhythm, as exemplified by the important insights provided by Phase Response Curve theory. However, if individual neurons fire irregularly (i.e., in the fluctuation-driven regime), and only take part in a subset of population cycles, the geometry of the regular spiking regime becomes less relevant, as neurons dwell most of the time in the subthreshold range.

In this study, we show how intrinsic neuronal oscillations at the subthreshold level affect the generation and properties of collective high-frequency oscillations. We focus on a regime where individual neurons fire irregularly at a rate that is considerably lower than the frequency of network oscillations. For simplicity, and considering the key role of synaptic inhibition in gamma rhythmogenesis, we consider purely interneuronal networks. Individual units receive spatially independent and noisy background inputs, thus mimicking an activated state of neuronal processing [46]. This is a key difference with respect to previous theoretical investigations on this topic, which poised individual neurons in the regular firing regime and neglected the effects of the strong barrages of background synaptic activity, which are expected to be prominent *in vivo*. As we will soon make clear, the influence of single-cell dynamics on network activity in a realistic context can only be thoroughly understood if the interplay with extrinsic inputs from other brain regions is taken into account.

Our network models can exhibit sinusoidal oscillations, as well as fully-blown, non-linear oscillations with highly synchronous firing. While we have studied a broad parameter space, our main focus is on the latter regime, as it more closely resembles the highly synchronous firing of basket interneurons during gamma-activated states in the cerebral cortex [19].

The presence of subthreshold oscillations affect several dimensions of single-cell dynamics. In response to a synaptic background bombardment, the restorative effect of a resonant current lowers firing rates and narrows the membrane potential distribution around the resting potential. Importantly, subthreshold oscillations change the functional coupling between neurons, i.e. the shape of post-synaptic potentials, and can result in post-inhibitory rebound depolarization. Some of these effects tend to enhance collective oscillations, while others tend to impair them. The adoption of neuron models with a fixed voltage threshold for spike generation enables us to disentangle these different effects. By independently varying the statistics of background inputs and the voltage threshold for spike generation, we can compare neuronal models that only differ along a single dimension of neuronal dynamics (e.g., in the presence of subthreshold damped oscillations, in conditions of equal firing rate response to the noisy background), hence elucidating the specific contribution of different features of single-cell dynamics that affect collective rhythmogenesis.

In this work, we adopt neuron models that linearly describe the subthreshold dynamics, where action potential generation is implemented via a voltage threshold-crossing reset: the Integrate and Fire (IF) and the Generalized Integrate and Fire (GIF). These neuron models only differ in their subthreshold dynamics, which is purely passive in the IF, while it exhibits subthreshold damped oscillations in the GIF. Importantly, they both exhibit type I PRC (inhibitory perturbations always result in phase delay) if made to fire regularly via the injection of a constant depolarizing curve (see section “Phase Response Curves in the IF and GIF neuron” and Figure S1 in Text S1). Correspondingly, when these neurons are coupled in a network by inhibition, the emerging collective dynamics only differ consistently when neurons are poised in the irregular, fluctuation-driven regime, but not when they are made to fire regularly in a mean-driven regime.

While 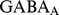 signalling is traditionally considered to be hyperpolarizing in the adult brain, it can be shunting or slightly depolarizing in some brain regions and neuron types [47], [48]. Shunting inhibition precludes post-inhibitory rebound depolarization. In these conditions the effects of firing rate and depolarization responses dominate the dynamics, yielding stronger oscillations in networks of purely passive neurons.

Synchrony and oscillations are dissociable concepts. Collective oscillations are possible in the absence of synchrony; for example, they can emerge as sinusoidal oscillations in the vicinity of a Hopf bifurcation [9]. In addition, synchronous firing can be observed in the absence of network oscillations, when neurons take part in population spikes that occur non-periodically [49]. In the networks considered in this study, synchronous collective oscillations are produced; hence, the two terminologies are used interchangeably.

## Methods

### Network Models

We consider a network of 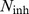 inhibitory neurons with all-to-all connectivity and equal weights. Neurons are placed on the vertices of a 2D uniform grid of size 1 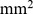 with periodic boundaries (a torus). Hence, every neuron is associated with a pair of values 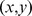, included in the unit square, denoting its relative spatial position. The distance 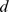 between a pair of neurons located at 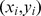 and 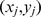 is calculated as
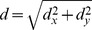
(1)where 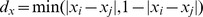 and 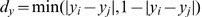. As opposed to excitatory connections, which decay with distance in probability and strength, inhibitory connections have been shown to be independent of distance in a small cortical patch [50]. Hence, in our models, synaptic weights 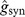 are equal for each pair of cells, and the topology is enforced by distance-dependent delays alone. Neuronal signals propagate with a conduction speed of 0.141 m/s, in accordance with experimental results in unmyelinated fibers supporting local, horizontal synaptic connections in cats and monkeys [51],[52]. 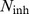 is taken to be equal to 400, which constitutes approximately 10% of the number of basket cells (an interneuronal type critically involved with high-frequency oscillations) in the dorsal hippocampus of the rat [53]. The adoption of a toroidal topology with all-to-all connectivity and equal synaptic strengths enables us to exploit the symmetry of the network and average the bivariate measures we consider over pairs of neurons separated by the same distance (see subsection “Measures”). This enables us to obtain precise estimates with reasonable computational cost. At the same time, both theoretical considerations as well as our own numerical simulations suggest that a network with sparse connectivity would yield the same qualitative results, because sparsity does not change the dynamic behavior of the network but just increases the level of finite-size effects (see, for example, [42]).

### Neuron Models

Individual neurons are described either as Integrate and Fire (IF) or Generalized Integrate and Fire (GIF). Both models adopt a linear description of the subthreshold dynamics, which is one-dimensional for the IF and two-dimensional for the GIF, and a threshold-based spike generation mechanism. The subthreshold dynamics are based on analogies with linear electric circuits (RC for the IF, RLC for the GIF), a formalism with a long and successful history in the phenomenological characterization of neuronal dynamics (for some early examples, see [54]–[57]; for a recent review, see [58], [59]). In the case of the IF, the voltage variable *v*, which measures the membrane potential deviation from the leak reversal potential, evolves according to the differential equation
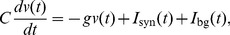
(2)where *C* is the membrane capacitance and *g* is the leak conductance. 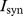 represents the inhibitory synaptic current resulting from action potential generation in other neurons of the network, and 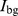 is a background term representing synaptic inputs from other brain areas not explicitly modelled. The subthreshold dynamics in the GIF includes an additional dynamical variable *w*, which represents the linearized effect of voltage-gated ion currents:
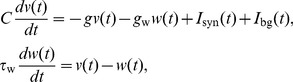
(3)where 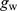 and 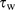 are the conductance and time constant associated with the *w* variable, respectively. The models are endowed with an after-spike reset mechanism, so that when *v* crosses a threshold 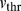 from below a spike is emitted, the membrane potential is reset to a value 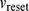, and kept there for a refractory time 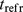. We set 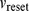 below the leak reversal potential, in accordance with the observation of after-hyperpolarization in PV basket cells [27]. Our canonical parameter set corresponds to a membrane time constant of 10 ms in both models, and a period of intrinsic subthreshold oscillations of ∼31 ms (∼32 Hz) in the GIF neuron, in accordance with the frequency of intrinsic subthreshold oscillations measured in fast-spiking inhibitory interneurons in the mammalian cortex and hippocampus (10–50 Hz [27], [30]). In the absence of external inputs, the IF responds to an instantaneous perturbation with an exponential relaxation to rest with rate *g*. Hence, it provides a simple description of purely passive subthreshold dynamics. In a certain parameter subspace (which includes the parameter set used here), the system (3) is characterized by a pair of complex conjugate eigenvalues (see the Appendix in Text S1). Therefore, the GIF neuron responds to perturbations with damped oscillations, and constitutes an analytically amenable model for the description of neuronal intrinsic oscillations, i.e., oscillations generated by intrinsic ionic mechanisms as the activation of a resonant current or the inactivation of an amplifying current [29]. These phenomenological models can be considered as linear approximations (one-dimensional in the case of the IF neuron, two-dimensional in the case of the GIF neuron) of more general neuronal models (see, for example, [35]). In fact, the only requirement that a detailed neuron model must satisfy for this approximation to be valid is the presence of a stable fixed point, where the Jacobian of the whole system is evaluated in order to obtain the coefficients of the corresponding IF or GIF description. This linear approximation is guaranteed to be valid in a small neighborhood of the stable fixed point. 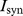 and 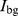 represent the synaptic current from other interneurons in the network, and the background input from other brain regions and other interneuronal types that are not explicitly modelled (e.g., somatostatin-positive Martinotti cells) and are described below. Parameter values and descriptions are provided in Table 1.

**Table 1:** Parameter descriptions and canonical values used throughout this study, unless otherwise stated.

Description	Parameter symbol and value
**Intrinsic parameters**	
membrane capacitance	*C* = 10 nF
leak conductance	*g* = 1 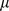 S
conductance associated with the *w* variable	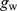 = 4 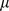 S
time constant of the *w* variable	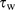 = 10 ms
threshold voltage	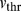 = 6.3 mV
reset voltage	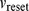 = 3 mV
refractory period	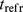 = 3 ms
**Coupling parameters**	
peak synaptic conductance	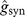 = 0.25 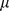 S
synaptic delay	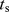 = 1 ms
propagation speed	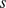 = 0.141 m/s
**Background input parameters**	
inhibitory reversal potential	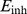 = −10 mV
excitatory reversal potential	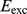 = 70 mV
inhibitory time constant	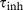 = 1 ms
excitatory time constant	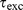 = 1 ms
mean BG excitatory conductance	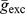 = 0.5 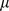 S
inh/exc mean conductance ratio	*k* = 5
BG excitatory conductance SD	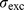 = 0.6 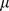 S
inh/exc BG conductance SD ratio	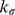 = 2.5

### Synapse Models

Synaptic coupling is described as
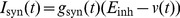
(4)

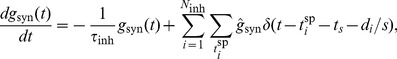
(5)where 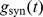 is the global inhibitory conductance impinging on the current neuron and 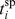 is the sequence of spike times generated by neuron *i*. Synaptic transmission is delayed by distance-dependent and distance-independent components 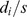 and 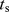, where 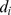 is the distance between the current neuron and neuron *i* (calculated according to (1)), *s* is the axonal propagation speed, and 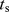 accounts for non-instantaneous processes at synaptic contacts. When a presynaptic pulse reaches the postsynaptic neuron, the synaptic conductance 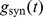 increases instantaneously by a value 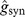, and then decreases exponentially to zero with time constant 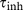. The corresponding synaptic current 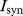 is then obtained by multiplication with the difference between the voltage variable and the reversal potential for inhibition 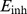. Unless stated otherwise, simulations are performed with the parameter values reported in Table 1.

### Background Noise

Every neuron receives a spatially independent background term 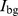, which is composed of an excitatory and an inhibitory component with associated reversal potentials 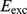 and 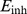:
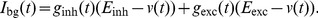
(6)


The background time-varying conductances are described as rectified Ornstein-Uhlenbeck processes with mean 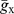, standard deviation (SD) 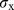, and autocorrelation time constant 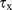 (x = inh, exc):
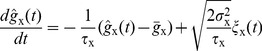
(7)

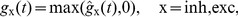
(8)where 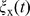 is Gaussian white noise with zero mean and unit standard deviation. We maintain a fixed ratio between the background inhibitory and excitatory conductance, both in terms of mean values and variability (unless stated otherwise). That is, 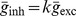 and 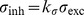. We choose *k* = 5 and 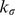 = 2.5 as canonical values, in accordance with *in vivo* estimates [60]. Isolated neurons respond to the synaptic bombardment with irregular firing at relatively high rates (GIF: 74 Hz, Inter-Spike Interval coefficient of variation (ISI CV) = 0.78; IF: 90 Hz, ISI CV = 0.81). Table 1 reports descriptions and canonical values for all parameters.

### Numerical Methods

Model equations (2) and (3) are integrated with a sixth-order, fixed step-size Runge-Kutta algorithm, with time step 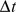 = 0.01 ms. The threshold-crossing and refractoriness conditions are evaluated only once per time step, as well as the calculation of synaptic currents according to equations (4) and (5). The background conductances 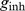 and 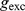 are updated at each time step using the properties of Ornstein-Uhlenbeck processes. That is, 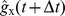 is normally distributed with mean 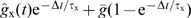 and standard deviation 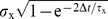. We initialize the networks randomly and discard the initial transient. We focus on the steady-state dynamics, that is, on the regime in which the statistical properties of network dynamics are time-invariant. This regime is typically reached within a few tens of milliseconds. However, transients can be longer for certain parameter sets that are close to the onset of collective oscillations. Hence, we discard the first 2 s of simulation time to ensure that any initial transient is excluded from the analysis.

### Measures

Neuronal network activities are quantified using several measures at the individual, pairwise and collective levels.

#### Single cell measures

The level of mean activation at the single neuron level is quantified with the firing rate 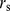, defined as the inverse of the mean Inter-Spike Interval (ISI). ISIs are obtained from the time ordered series of spike event times 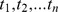 as 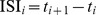, 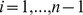. Spiking irregularity is measured with the coefficient of variation (CV) of ISIs, defined as the ratio between the standard deviation (SD) of ISIs and their mean value. Subthreshold activity is characterized by estimating the probability density function (pdf) of the membrane potential variable *v* and its mean value.

#### Mean phase coherence

We quantify phase relationships between firing activity in different cells by calculating the Mean Phase Coherence (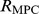) between every pair of neurons [61]. The Mean Phase Coherence between neurons *A* and *B* is a complex number defined as
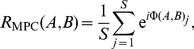
(9)where *S* is the number of spike events in neuron *A* that occur within an ISI in neuron *B*, and 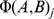 is the phase of spike 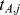 in neuron *A* with respect to the smallest ISI in neuron *B* that contains 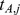. That is, if 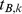 is the largest spike time in *B* smaller than 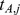, and 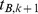 is the smallest spike time in *B* greater than 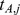, 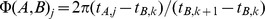. Because of toroidal symmetry, there is no reason for a given neuron to lead ahead or lag behind another neuron on average. The only non-trivial phase relationships that can be established are in-phase or anti-phase. Hence, 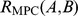 is a real number, positive (negative) if in-phase (anti-phase) firing is preferred. However, its numerical estimation is complex valued. In order to reduce the error in the estimation of the Mean Phase Coherence, we exploit the toroidal symmetry of the network and average 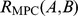 over neuron pairs with a given distance. In order to yield the same number of pairs for every value of *d*, only values of *d* that are multiple of the distance between adjacent neurons in the *x* or *y* direction are considered:
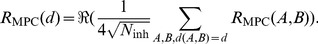
(10)


The global level of phase coherence is quantified by
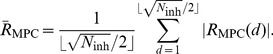
(11)


#### Population measures

The global, time-varying level of spiking activity in the network is measured by counting the number of spikes occurring in 10 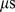 time bins. We refer to this measure as Population Rate (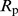).

In order to assess the state of the network at the subthreshold level, we calculate, in each 100 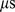 time bin, the mean value across neurons of the membrane potential *v*, the intrinsic current 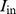 (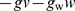 for the GIF, 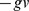 for the IF), and the synaptic current 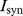. We divide the time series into non-overlapping windows of 20 ms duration (corresponding to approximately two oscillation cycles), and independently fit each short series with a sinusoidal function 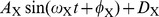, 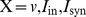. Histograms of the root mean squared error (for each variable) and of the angular frequency (pooling the three variables together) show inflection points, which are used to select only those time windows where the time evolution of the mean 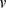, 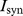, and 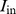 could be properly described by a sinusoid. Fitted traces are further inspected visually to ensure stationarity and proper fitting in each time window. Time windows that do not comply with these requirements are excluded from the analysis. This procedure removes a different number of time windows for each network considered, ranging from 9% to none.

Phase values for the intrinsic and synaptic mean currents with respect to the voltage oscillation are calculated in each time window as 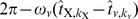, where 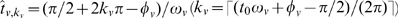 is the time of the first peak of the sinusoidal fit to the membrane potential in the current time window, which starts at 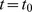, and 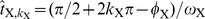 (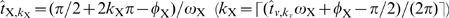) is the time of the first peak of the sinusoidal fit to the current X which follows 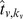 (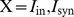), and mean phase relationships are calculated. In order to investigate the relationships between oscillation strength and the phase of intrinsic and synaptic currents in each time window, we calculate *circular-linear* correlations, namely between phase values (circular variables) and corresponding 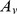 values (linear variables). The amplitude of the sinusoidal fit to the mean membrane potential oscillation 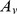 can be considered as a temporally local measure of synchrony. We also calculate p-values of the null hypothesis of no correlation, using standard circular statistics [62] as implemented in the CircStat toolbox for MATLAB [63].

Further information is obtained by estimating the full probability density function (pdf) of the intrinsic current 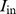. Time windows corresponding to refractory periods are removed, and probability densities are estimated using either all remaining data points (unconditional pdfs), or only those that fall around the peaks or troughs of the oscillation (phase conditioned pdfs, half-width = 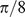 radians), as identified by sinusoidal fits to short traces of the average 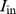 across neurons. Bivariate probability densities 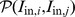 are estimated with symmetric Gaussian kernels for pairs 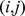 of adjacent neurons. The deviation from independence of the 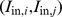 distribution is measured as the difference between the estimated bivariate pdf and the pdf that would be expected if 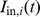 and 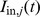 were independent processes: 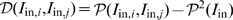. As in the univariate case, bivariate pdfs and corresponding deviations from independence are measured both unconditionally and conditioning on the phase of the average 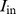 across neurons.

We also estimate bivariate probability densities of the mean and standard deviation (across neurons) of the membrane potential variable. In this case, the variances along the two dimensions are not expected to be equal, hence an adaptive kernel density estimator based on linear diffusion processes is used [64]. We use a perceptually balanced colormap with cubic-law luminance values in order to avoid perceptual biases induced by standard rainbow colormaps (pmkmp.m, available from http://www.mathworks.com/matlabcentral/fileexchange/28982-perceptually-improved-colormaps, last accessed March 2013).

#### Spectral analysis

We used the Chronux data analysis toolbox (http://chronux.org) for the spectral analysis of model data [65]. We estimated the collective oscillations produced by our networks by computing the power-spectra of 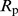. The frequency of network oscillations 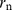 is quantified as the maximum of a Gaussian function fitted to the power spectrum of 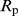.

## Results

### Single Neuron Dynamics

Before we consider the dynamics of networks of neurons coupled by inhibitory conductances, it is instructive to characterize how individual neurons respond to the background noisy input alone, which represents input from other brain areas and is the main source of depolarization and variability in the model. Hence, we performed a linear analysis of the eigenvalues of the model neurons with fixed external conductances (with 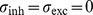).

**Figure 1:**
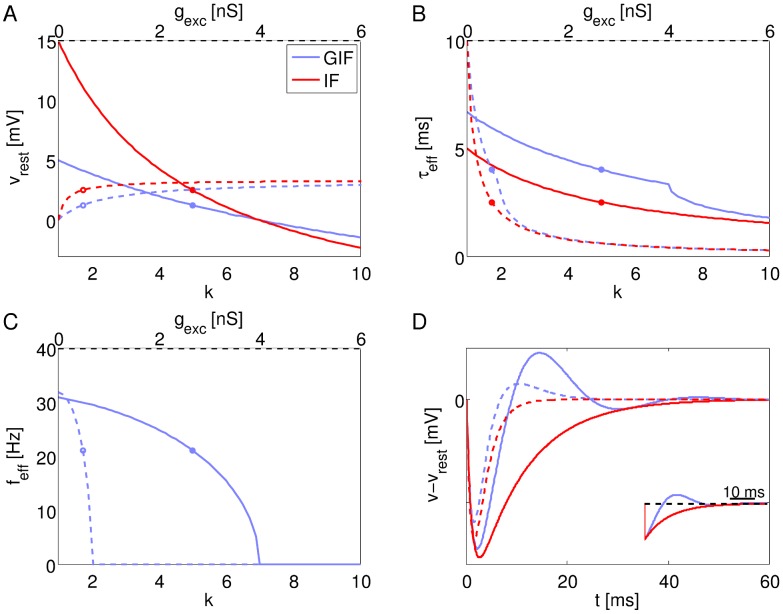
Linear analysis of the neuron models. Linear analysis of the model neurons with fixed external conductances. A: Resting potential as a function of the background inhibition-to-excitation ratio *k* for the canonical value of 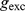 (solid lines), and as a function of the background synaptic excitation 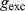 for the canonical value of 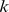 (dashed lines). Solid lines refer to the bottom axis; dashed lines to the top axis. Circles show the canonical values of the corresponding parameters. B: Effective membrane time constant as a function of *k* and 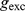. Line styles, colors and symbols as in A. C: Effective intrinsic frequency as a function of *k* and 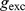. Line styles, colors and symbols as in A. D: IPSPs in response to a single presynaptic pulse delivered at time t = 0, in the presence (dashed lines) or absence (solid lines) of fixed background conductances. Inset shows the membrane potential response to an instantaneous perturbation.

Figure 1 shows the effect of the background conductance 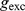 on the resting potential, effective membrane time constant and effective intrinsic frequency for the canonical ratio between background inhibition and excitation (*k* = 5), and the effect of the background inhibition-to-excitation ratio *k* for the canonical value of the background excitatory conductance (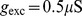) on these same quantities. The resting potential is defined as the voltage satisfying 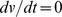 in equations (2) (IF) or (3) (GIF), while the effective membrane time constant 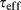 and the effective intrinsic frequency 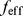 are defined from the eigenvalues 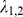 of the systems (2) or (3) as 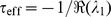 and 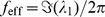, respectively. Full expressions are reported in the Appendix, Text S1.

When background inhibition and excitation are balanced at the canonical ratio *k* = 5, the resting potential is above zero but below threshold even for very large values of the background conductance 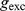 (Figure 1A). In this regime, spiking is irregular and is induced by random fluctuations in the background conductances. The injection of background noisy conductances mimics an activated state of the neuronal microcircuit, and decreases the effective membrane time constant, as previously reported [66], [67] (Figure 1B). In addition to this, we observe a decrease in the resonant frequency with increasing background drive in the GIF, which eventually results in a purely passive dynamics for 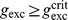 (Figure 1C) as the canonical GIF eigenvalues coalesce onto the real axis.

Varying the background inhibition-to-excitation ratio *k* shifts the resting potential and the membrane potential distribution (Figure 1A, see also Figure 5A). The shift is greater in the IF neuron because the additional dynamical variable in the GIF counteracts voltage changes away from zero. If all the other parameters are held fixed, the depolarization of the membrane potential distribution induced by a decrease in *k* also translates to an increase in the mean firing rate. However, the adoption of neuron models with a fixed voltage threshold for spike generation enables one to adjust the threshold in order to maintain a desired rate of firing for each value of *k* and for both neuron models considered.

As a consequence of the changes in membrane time constant and oscillation frequency, Inhibitory Post-Synaptic Potentials (IPSPs) in the presence of constant background conductances are smaller and narrower (Figure 1D). In the case of the GIF, intrinsic oscillations are more strongly damped and exhibit a lower frequency.

### Network Dynamics

We consider the activity generated by a network of identical spiking neurons with all-to-all inhibitory connectivity. Coupling is delayed by a distance-dependent component, mimicking axonal propagation delays, and a distance-independent component that accounts for non-instantaneous dynamics at synaptic contacts. In addition to inhibitory conductances elicited by action potentials generated in their peers, individual neurons also receive random and spatially independent background synaptic conductances. In the absence of coupling, the background synaptic bombardment sets the neurons in an irregular firing regime.

In a broad range of parameter space, network oscillations are produced at high frequency (∼100 Hz). As inhibitory coupling and background inhibition-to-excitation ratio are varied, oscillation strength and single-cell firing rates are modulated. However, the frequency of network oscillations is only slightly affected, with stronger coupling and lower depolarization resulting in slower collective oscillations, as previously reported in modelling and experimental studies [7], [68].

While we have varied most parameters in ranges that are in accordance with physiological data, we choose a representative parameter set that corresponds to fully developed, yet unsaturated, oscillations in the GIF network. The results we report in this section refer to the canonical parameter set, while the effects of variations in the inhibitory coupling and/or in the background inhibition-to-excitation ratio *k* are reported in section “Effects of rate, membrane potential distribution and coupling on synchrony”.

**Figure 2:**
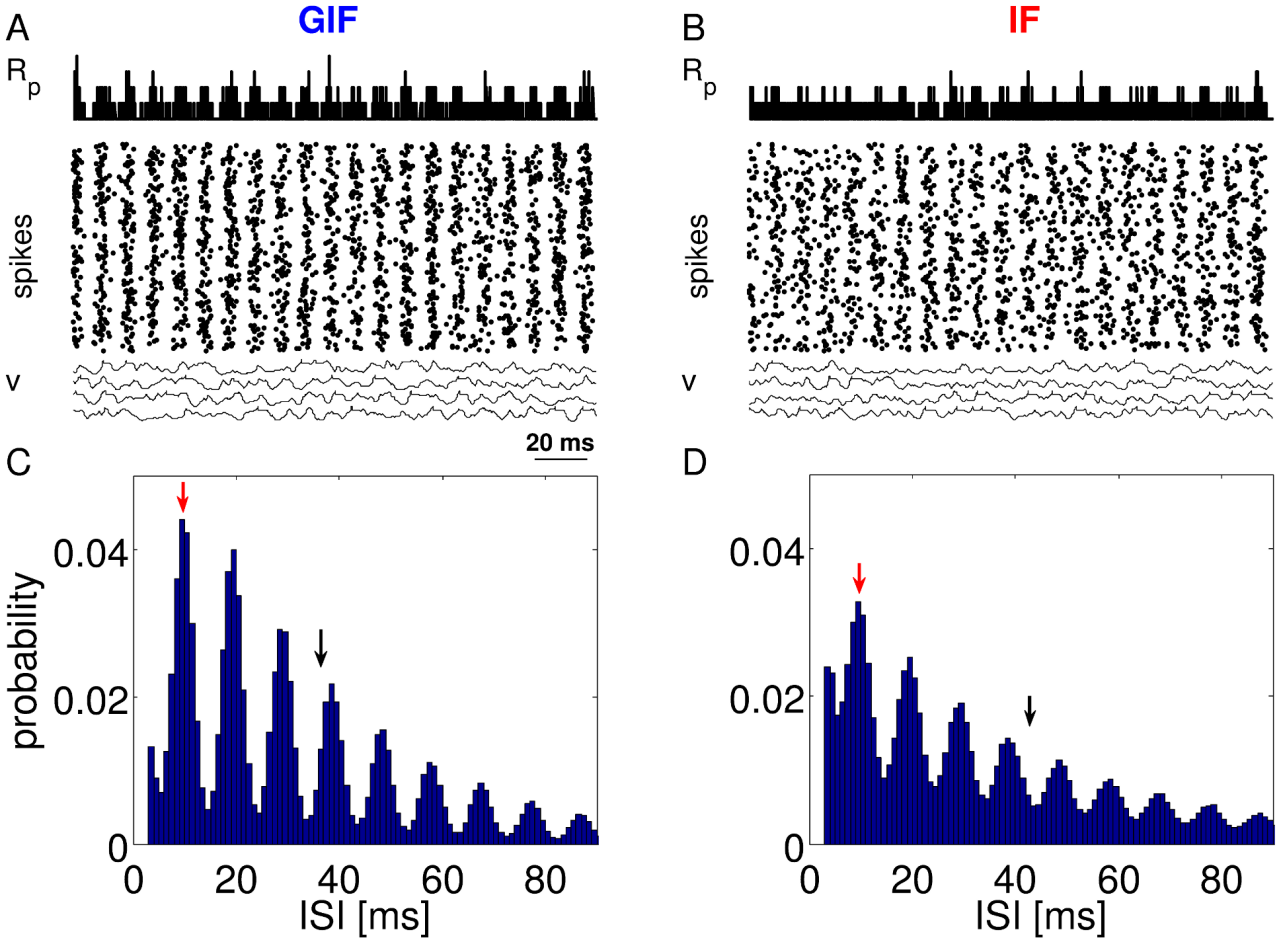
Sample activity in the canonical GIF and IF networks. A: Population rate (top), spike raster plot (middle), and *v* trajectories of selected neurons (bottom) in the GIF network for a representative parameter set. C: Single-cell ISI histogram for the GIF network. The red arrow indicates the period of network oscillations. The black arrow indicates the mean single-cell ISI. B, D: The same as A and C, for the IF network. Oscillations are more prominent in the GIF network.

Figure 2 shows representative activities generated by a network composed of GIF neurons (left panels), or IF neurons (right panels). It is apparent from both the 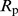 traces (top rows) and the raster plots (middle row) that the GIF network exhibits more prominent oscillations (quantified in Table 2). In this network, oscillations are fully developed and there are narrow temporal windows in between volleys of activity during which almost no spike is produced. The IF network also produces oscillations, but in this case, the firing probability in between peaks of activation does not completely vanish. Membrane potential trajectories of individual neurons are also more strongly correlated in the GIF network, with downward deflections corresponding to peaks of inhibitory drive showing greater correspondence across cells.

**Table 2:** Single-neuron and network statistics for the IF and GIF models considered.

	IF	GIF	IF r-matched	GIF r-matched
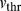	6.3	6.3	7.3	5.5
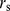 (Hz, 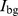 only)	90.3	73.7	73.8	89.5
ISI CV ( 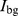 only)	0.81	0.78	0.83	0.76
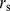 (Hz)	23.3	27.4	19.7	32.9
ISI CV	0.94	0.84	0.95	0.8
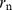 (Hz)	103.1	103.6	101.4	104.5
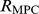	12.8 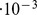	25.4 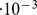	7.3 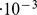	40.4 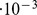

The first row shows the voltage threshold for spike generation 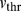, which has been adjusted to yield approximately the same firing rate response to the background synaptic bombardment in canonical and r-matched neurons. The second and third rows show the single-neuron statistics to the background synaptic bombardment alone. The fourth and subsequent rows show single-neuron and ensemble statistics measured in network simulations. 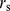, single-neuron firing rate; ISI CV, coefficient of variation of inter-spike intervals; 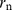, frequency of network oscillations; 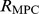, mean phase coherence.

Individual neurons fire irregularly at a rate that is much lower than the frequency of collective oscillations. As shown in Figure 2C and D, single-neuron ISI histograms are multipeaked. The first peak corresponds to the population period, and lumps the contribution of pairs of spikes emitted by the same cell in adjacent cycles. Subsequent peaks are gradually smaller and occur at integer multiples of the population period. The mean ISI is about 4 times greater than the population period. The envelope of the distribution resembles an exponential distribution, a signature of irregular, Poisson-like spiking.

The periodic modulation of excitability is more prominent in the GIF network, where the ISI probability vanishes almost completely in between peaks. A small peak is discernible at very short ISIs, just longer than the refractory period. This peak lumps the contribution of spike doublets emitted by the same cell in the same cycle. These events are much more likely in the IF network, where oscillations are not fully developed and inhibitory volleys are not strong enough to completely preclude spiking during the inactive phase of the oscillation.

The higher synchrony exhibited by the GIF network corresponds to higher firing rates: just before the onset of a population spike, inhibition vanishes almost completely, and this allows for a greater number of neurons to reach threshold and take part in the population spike. Conversely, in the IF network, there is a residual amount of inhibition that is present even at the trough of inhibitory volleys. This tonic component results in a smaller number of cells taking part in the population spike.

The inclusion of an additional dynamical variable *w* in the GIF model, which implements a restorative force on the membrane potential dynamics, induces several changes in the neuronal response to background synaptic bombardment or isolated synaptic potentials. The dynamical variable *w* counteracts voltage changes, hence the GIF neuron exhibits a narrower membrane potential distribution, lower firing rates and lower firing variability. Firing rates have a strong influence on the level of synchronization that can be achieved in the coupled network, as higher firing rates induce inhibitory conductances of greater amplitude that more effectively drive the membrane potential near the reversal potential of inhibition at each inhibitory peak of the oscillation.

In order to elucidate the influence of firing rate changes in the observed differences in synchronization between GIF and IF networks, we considered an additional pair of models: *r-matched* GIF and *r-matched* IF. The r-matched GIF (r-matched IF) neuron is equal to the GIF (IF) neuron, except for the voltage threshold for action potential generation, which has been adjusted in order to yield the same firing rate response as the IF (GIF) neuron to the synaptic background bombardment.

**Figure 3:**
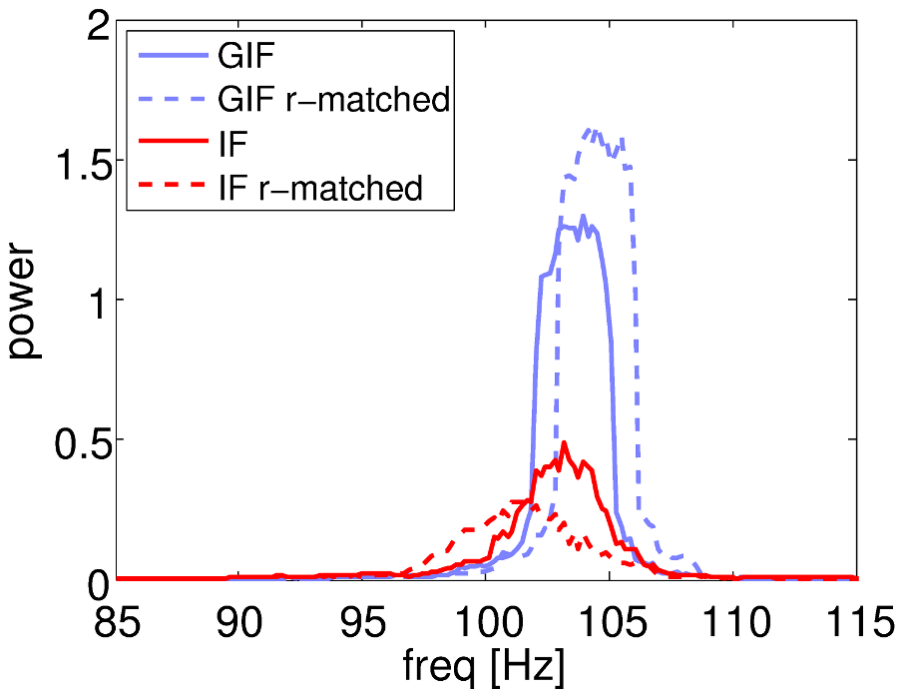
Power spectrum of population activity. Power spectrum of the population rate 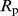 for the four model networks considered. Population activity exhibits a peak at ∼100 Hz, with higher synchrony corresponding with slightly higher population frequency. Note the tall, non-Gaussian peaks in the GIF networks, corresponding to non-linear, fully developed oscillations. In contrast, power spectrum peaks in the IF networks are bell-shaped, a signature of sinusoidal oscillations.

As shown by a power spectrum analysis of population activity (Figure 3), GIF neurons synchronize more *in spite of* the lower firing rate response to the noisy background exhibited by this model. In fact, the r-matched GIF, which displays the same firing rate response to the background as the IF, exhibits even stronger oscillations. Likewise, the r-matched IF neuron, whose firing rate response has been decreased to match the GIF, synchronizes more weakly than the canonical IF. Oscillation frequency depends only weakly on the synchronization level, with higher synchrony corresponding with faster oscillations.

Single-cell firing statistics and network oscillation measures for the four model networks considered are reported in Table 2.

## Distance Modulates Firing Synchrony and Subthreshold Correlations

The adoption of a network model with spatial extension enables one to study the spatial modulation of synchrony in the network activity. From a theoretical perspective, we expect that spatial modulation will be affected by two opposing influences. Neurons that are located nearby will experience similar patterns of incoming PSPs, because the coupling delays from any other neuron in the network will be similar. This is expected to increase synchrony among local pairs of neurons. However, nearby neurons are connected by rapid inhibition with short propagation delays. Hence, if the propagation delays among adjacent neurons are shorter than the temporal width of a population spike, this will tend to decrease synchrony among local pairs of neurons. In order to quantify the spatial structure of correlations in the inputs that neurons receive, in their internal states, and in the outputs they emit, we calculate average Pearson correlations between synaptic inputs, membrane potential variables, and mean phase coherence among neuron pairs as a function of their distance in the cortical sheet.

**Figure 4:**
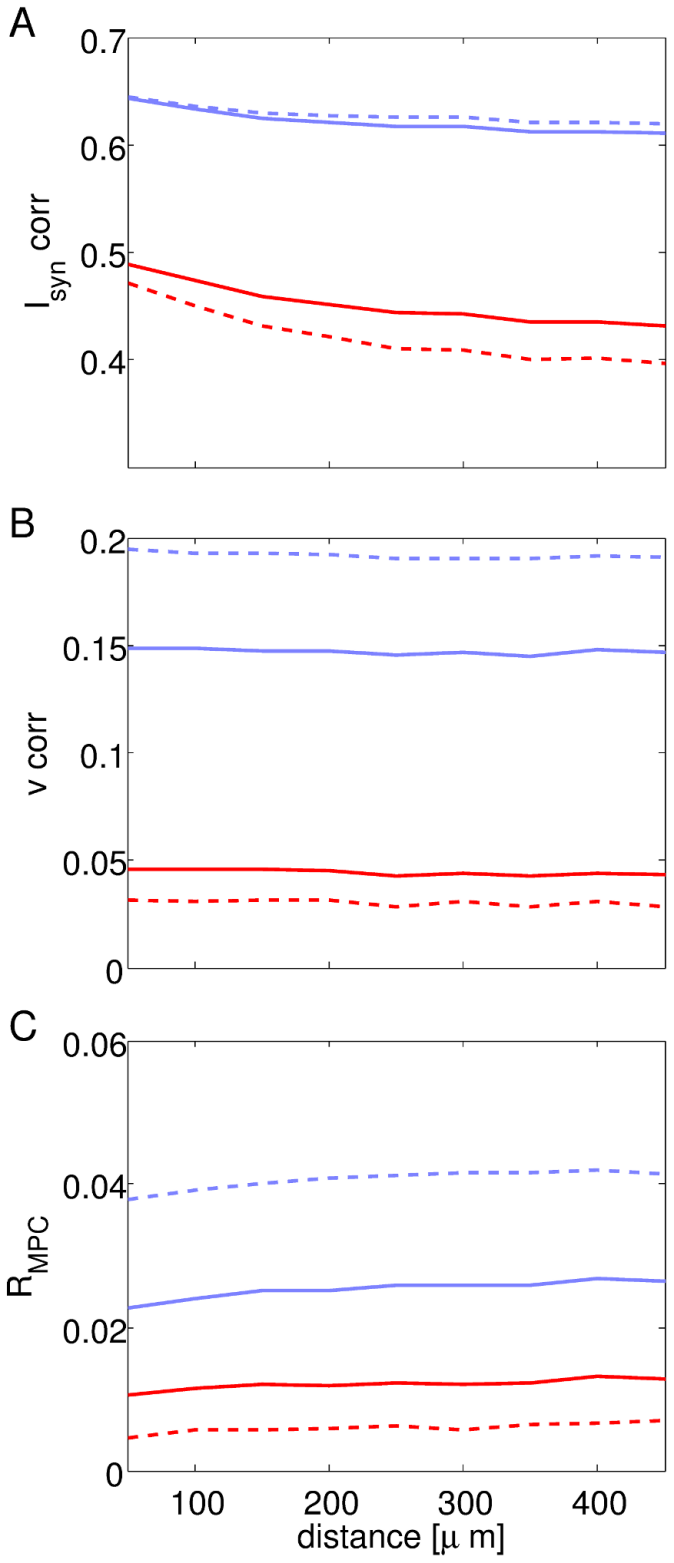
Correlation between synaptic inputs, membrane potential trajectories and *R*_MPC_ among pairs of neurons as a function of distance. A: Average Pearson correlation between incoming synaptic currents to neuron pairs as a function of their distance. Note the high correlation for nearby neurons, which decreases with distance. B: Average Pearson correlation between the membrane potential of neuron pairs as a function of their distance. The modulation with distance is negligible. C: 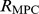 between firing patterns of neuron pairs as a function of their distance. 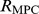 decreases at short distances due to short-latency mutual inhibition. Line styles and colors as in Figure 3.

As shown in Figure 4A, the correlation between synaptic currents to neuron pairs decreases as a function of distance. This is a result that we expect from the topological structure of the network. However, at the level of the membrane potential, correlations between neuron pairs are independent of distance (Figure 4B). This apparent incongruence is resolved if one takes into account the de-synchronizing effect of short-latency mutual inhibition between neurons. In fact, at the suprathreshold level, the synchronous firing of neuron pairs (as measured by 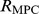) decreases at short distances (Figure 4C).

**Figure 5:**
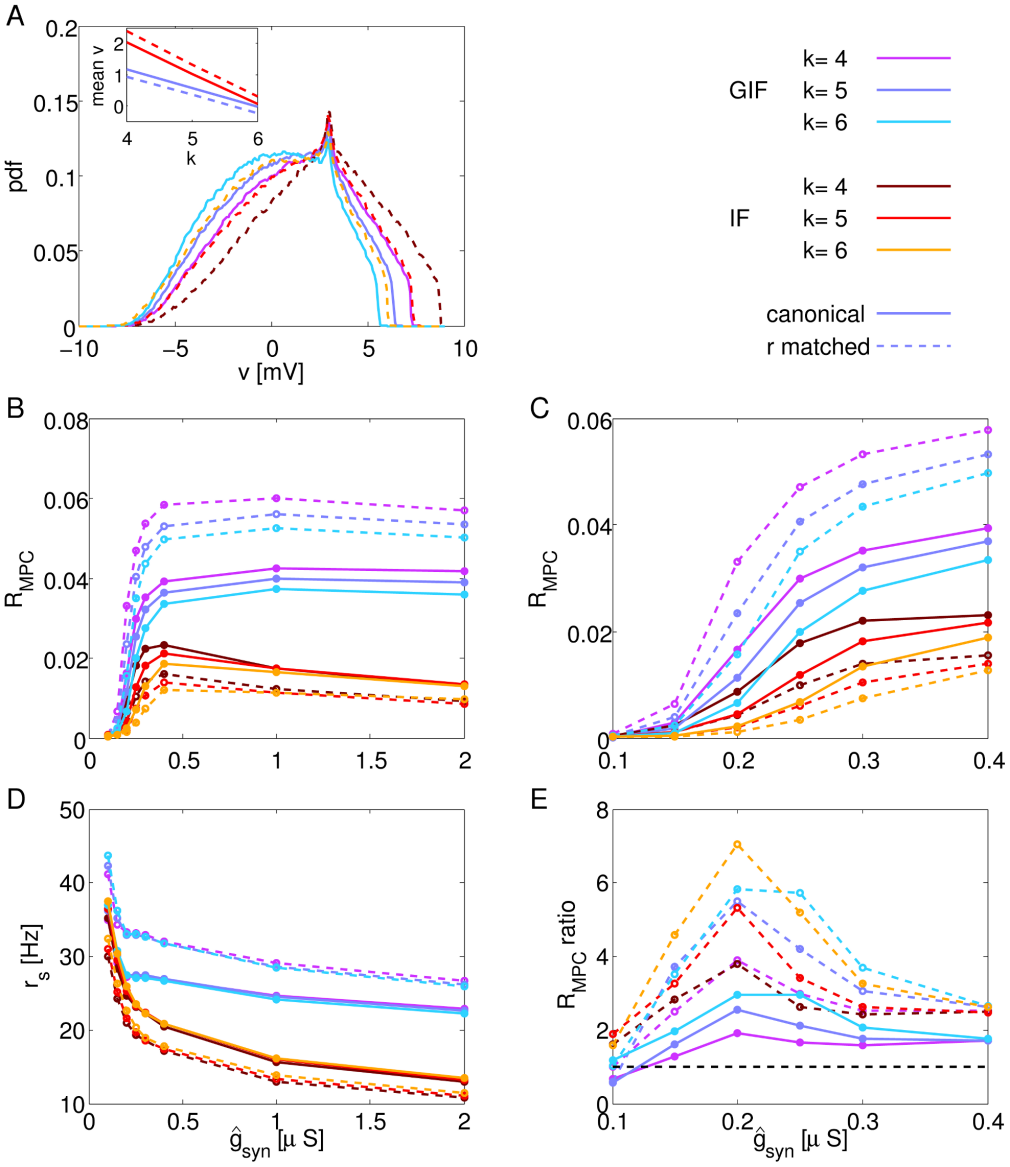
Effects of membrane potential depolarization and coupling on network dynamics. A: Membrane potential distributions of GIF and r-matched IF neurons in response to the background input alone, for different values of the background inhibition-to-excitation ratio *k*. In all cases, the voltage threshold for spike generation 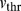 has been adjusted in order to match the rate response of the canonical GIF (73.7 Hz). Color and line style code as indicated. Inset shows the mean membrane potential as a function of *k* for the four model networks shown in Figure 3 (same line colors and styles). B, D: Synchrony (as assessed by 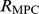, B) and firing rates (D) as a function of coupling strength 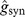. C: Enlarged view of B for low values of coupling strength. E: Ratio between corresponding curves in C. Solid lines show ratios between canonical models (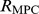
(GIF)/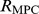
(IF)), while dashed lines show ratios between models that have been adjusted in order to exhibit the same rate response to the background input (purple, blue and light blue, 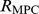
(GIF)/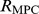
(r-matched IF); brown, red and orange, 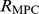
(r-matched GIF)/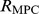
(IF)). Dots in B–E indicate simulated points, lines are drawn to guide the eye.

This phenomenon highlights a novel aspect of pattern decorrelation by inhibitory feedback [69], namely that rhythmic mutual inhibition with topologically structured delays can offset the spatial bias of incoming synaptic inputs and yield a flat profile of membrane potential correlations. Strong interneuronal oscillations drive the membrane potential of all neurons to a narrow range near 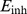 at the peak of the inhibitory drive in each cycle, so that the identity of the neurons that will be active in each cycle will faithfully represent the spatial pattern of inputs and will not be significantly biased by topological aspects. This property allows network activity to closely follow cycle-to-cycle variations in the spatial patterns of incoming stimuli [70].

## Effects of Rate, Membrane Potential Distribution and Coupling on Synchrony

In previous sections, we showed that intrinsic subthreshold oscillations enhance high-frequency oscillations, and that this effect occurs *in spite of* the reduction in firing rate due to resonant intrinsic dynamics. If the voltage threshold for spike generation 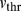 is differentially adjusted in order to yield the same mean rate response to synaptic background inputs in the passive and resonant neuron, the oscillatory advantage of GIF networks is even more prominent.

Other factors being equal, an increase in firing rates is expected to enhance collective oscillations. The more cells take part in the active phase of the oscillation, the greater the inhibitory synaptic conductance impinging on each neuron will be, thus driving the membrane potential to a more narrow range near the inhibitory reversal potential 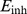. This effect has been described previously by others (e.g., [9]). However, most previous studies on high-frequency oscillations that adopted the IF formalism considered current-based coupling (but see [10]). This approximation is convenient for obtaining analytical results that relate the amount of synchronization to microscopic quantities such as single-cell firing rates, but can result in artificial dynamics where the membrane potential of individual neurons can fall to unrealistically hyperpolarized values.

The inclusion of more realistic conductance-based coupling reveals an additional factor that modulates the strength of oscillations produced by an interneuronal network: the relationship between the membrane potential distribution and the inhibitory reversal potential 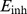. As the difference between the membrane potential distribution and the inhibitory reversal potential increases, the driving force of inhibition (i.e., 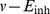) will increase. Hence, inhibition will be more effective at driving the membrane potential of individual neurons to a small region near 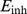 at each peak of the inhibitory drive, thus delivering a more efficient reset and strengthening the overall coherence of emerging oscillations.

In this section, we vary the background inhibition-to-excitation ratio *k*, and consider a depolarized set of networks (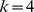, purple curves for the GIF, brown curves for the IF in Figure 5), a hyperpolarized set of networks (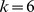, light blue curves for the GIF, orange for the IF), along with the canonical set of networks (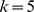, blue curves for the GIF, red for the IF). In all networks, single-cell firing rate responses to the background input have been adjusted to be equal (up to ±1% tolerance) to the canonical GIF rate response (74 Hz, GIF and r-matched IF) or to the canonical IF rate response (90 Hz, IF and r-matched GIF). Figure 5B–D shows the degree of synchronization and firing rates in these networks as the inhibitory coupling 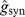 is varied.

As coupling is increased from zero, the network dynamics shift from asynchronous activity (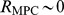) to fully developed oscillations, which saturate at 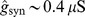. In this regime, neurons are reliably driven to a narrow range near 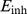 at each peak of the inhibitory drive. Further increases in 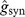 do not result in enhanced synchrony; on the contrary, they can result in a residual amount of inhibition during the active phase of the oscillations, which decreases firing rates and slightly decreases synchrony (Figure 5D and B). These effects are stronger in IF networks, as post-inhibitory rebound in GIF neurons diminishes the silencing effect of inhibition.

For all values of coupling, GIF neurons synchronize more, and that happens *in spite of* the reduction in rate response to the background input. In fact, r-matched GIF networks exhibit even stronger synchronization (purple, blue and light blue dashed lines in Figure 5B and C), while r-matched IF networks synchronize even less than canonical IF networks (brown, red and orange dashed lines).

Not surprisingly, increased inhibitory coupling decreases single-cell firing rates (Figure 5D). Even if GIF and r-matched IF neurons have been adjusted to exhibit the same firing rate response to the background input, the collective dynamics they exhibit as a population when connected through inhibition is the dominant effect on the resulting firing rates, even at low coupling values. Higher synchrony (r-matched GIF, GIF) corresponds with higher firing rates, as phasic inhibition allows for “windows of opportunity” for spiking activity. Conversely, in lower synchrony networks (r-matched IF, IF), inhibition has a tonic component that only allows action potential generation in those few cells that receive strong depolarization.

While the firing rate during network simulations depends on recurrent interactions within the local network, the firing rate in response to the noisy background is an intrinsic property of the neuron (given the statistics of the noisy background), and can be considered equivalent to the excitability or propensity to fire of the neuron. The relationship between synchrony and firing rate during network simulations is circular: higher synchrony results in phasic inhibition, allowing for greater “windows of opportunity” for spiking activity, while at the same time higher firing rates induce inhibitory conductances of greater amplitude that more effectively drive the membrane potential near the reversal potential of inhibition at each inhibitory peak of the oscillation. Conversely, higher firing rate responses to the noisy background causally result in higher synchrony for a given neuron type and inhibition-to-excitation ratio *k* (Figure 5B and C, compare solid and dash lines of the same color. Dash lines correspond to higher firing rate response to the noisy background for the GIF neuron, while the opposite holds for the IF neuron, as explained when introducing the *r-matched* models in section “Network dynamics”).

Importantly, the measure of synchrony that we use, Mean Phase Coherence (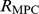, defined by equation (9)), does not have a built-in dependency on firing rate. In fact, 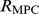 measures the level of phase (rather than time) coherence. Hence, an increase in firing rate results in lower values of 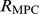 for the same level of coherence in time. Therefore, the relationship we describe between synchrony and firing rates during network simulations is, *a priori*, unexpected, and derives from the mutual dependence between single-neuron activity and network dynamics.

As expected from the argument exposed above, membrane potential depolarization is an additional factor in determining the strength of collective oscillations. More depolarized networks (purple for the GIF, brown for the IF) synchronize more effectively than their hyperpolarized counterparts (light blue for the GIF, orange for the IF).

As shown in Figure 5C and E, the oscillatory advantage of GIF networks is more prominent at intermediate values of the inhibitory coupling, with the 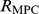 ratio between GIF and IF networks that peaks at 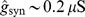. Here, the canonical GIF networks synchronize ∼3 times stronger than the canonical IF networks (as assessed by 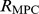), with that ratio increasing to ∼7 if networks with the same rate response to the background input are compared. The non-monotonic dependence of the 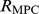 ratio on coupling shows that the synchronizing effect of inhibition becomes effective at much lower values of 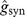 in GIF networks.

### Intrinsic Mechanisms that Enhance Collective Oscillations

We have shown that neurons with subthreshold oscillations synchronize more strongly than passive neurons when coupled by inhibition. However, they also exhibit lower firing rates and less depolarization in response to the background input, and both effects weaken collective oscillations. Hence, the question arises as to what are the intrinsic dynamical mechanisms that enhance oscillations in GIF neurons, in spite of the relative disadvantage resulting from their lower rate and depolarization responses.

In this section, we perform a detailed analysis of the intrinsic and synaptic currents flowing through the neuronal membrane at different phases of the oscillation cycle, and show that the synchronization advantage of GIF neurons can be understood as a result of the strong and coherent activation of inward intrinsic currents near the trough of membrane potential oscillations.

**Figure 6:**
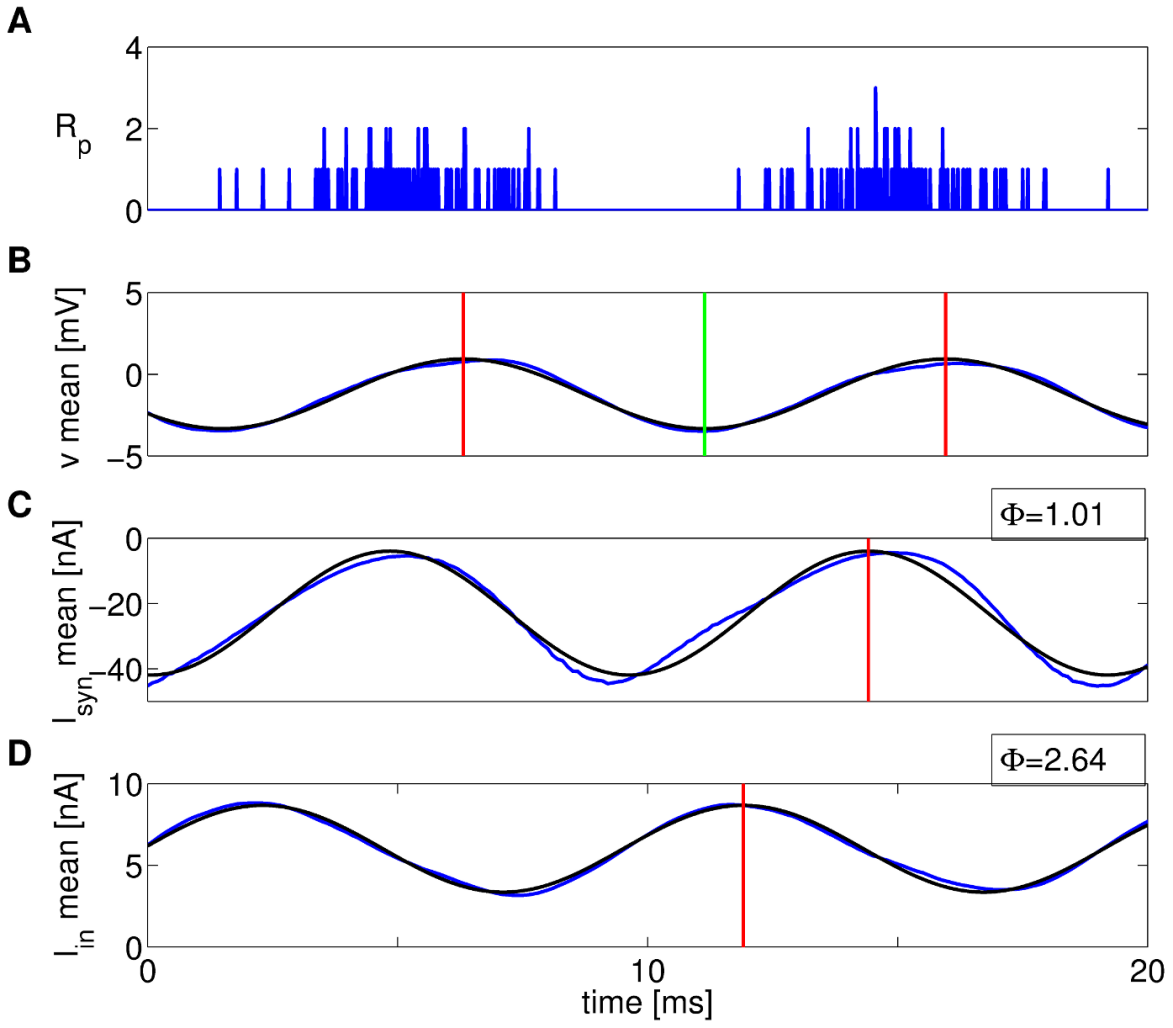
Intrinsic and synaptic currents. Population rate 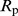 (A), along with the mean membrane potential *v* (B), the mean synaptic current 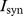 (C), and the mean intrinsic current 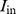 (D) across neurons for the GIF network in a short representative time window. Blue lines show simulation results, black lines are least-squares sinusoidal fits. The red vertical lines indicate the peak of the sinusoidal fit to the corresponding traces, the green vertical line indicates the trough of the sinusoidal fit to the mean membrane potential. Phase leads (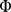, in radians) with respect to the mean membrane potential oscillation are shown for the mean synaptic current 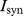 and the mean intrinsic current 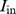.

Figure 6 shows the GIF population rate 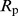, along with the membrane potential *v*, the synaptic current 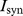, and the intrinsic current 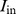, averaged across neurons, for a few oscillation cycles. The intrinsic current is equal to 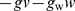 for the GIF, and to 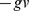 for the IF (see equations (3) and (2)). In each cycle, the average membrane potential reaches a peak near the end of the population spike. About a third of a cycle later, the average inhibitory synaptic current reaches a minimum, which then results in a minimum of the average membrane potential as neurons are driven to a small range near the reversal potential of inhibition. After a small lag corresponding to the time scale of subthreshold neuronal dynamics, intrinsic currents peak, facilitating the recovery from inhibition of the average membrane potential, which in turn leads to the next active phase of the oscillation.

**Figure 7:**
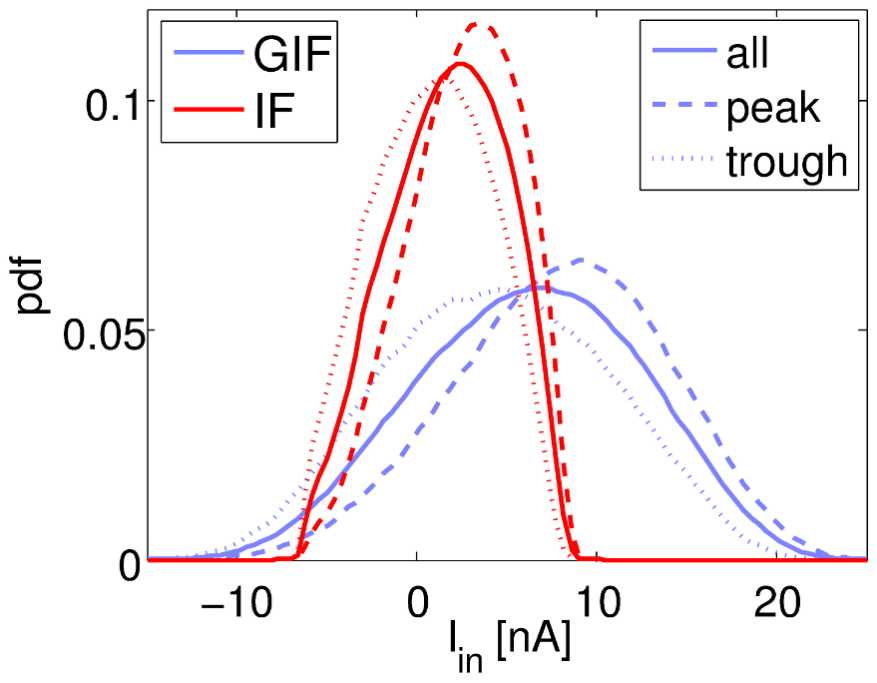
Distribution of intrinsic currents conditioned on the phase of the population rhythm. Probability density functions of intrinsic currents 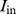 in the GIF (blue) and IF (red) networks. Solid lines indicate unconditional probability densities, dashed (dotted) lines indicate probability densities conditioned on the peak (trough) of the mean 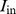 oscillation.

We reasoned that if inward currents are stronger in GIF neurons near the trough of the membrane potential oscillation, that would constitute a depolarizing force, coherent across neurons, that acts selectively in the later portion of the inactive phase of the oscillation, hence constituting a candidate for a synchronization mechanism. In order to assess the contribution of intrinsic currents to the generation of synchronized oscillations, we estimate the probability density 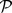 of the intrinsic currents in the GIF and IF networks (Figure 7). We estimate 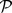 by using either all available data points (solid traces), or only those data points that fall around the peaks or troughs of the oscillation, as identified by sinusoidal fits to short traces of the average 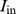 across neurons (dashed and dotted lines, respectively).

**Figure 8:**
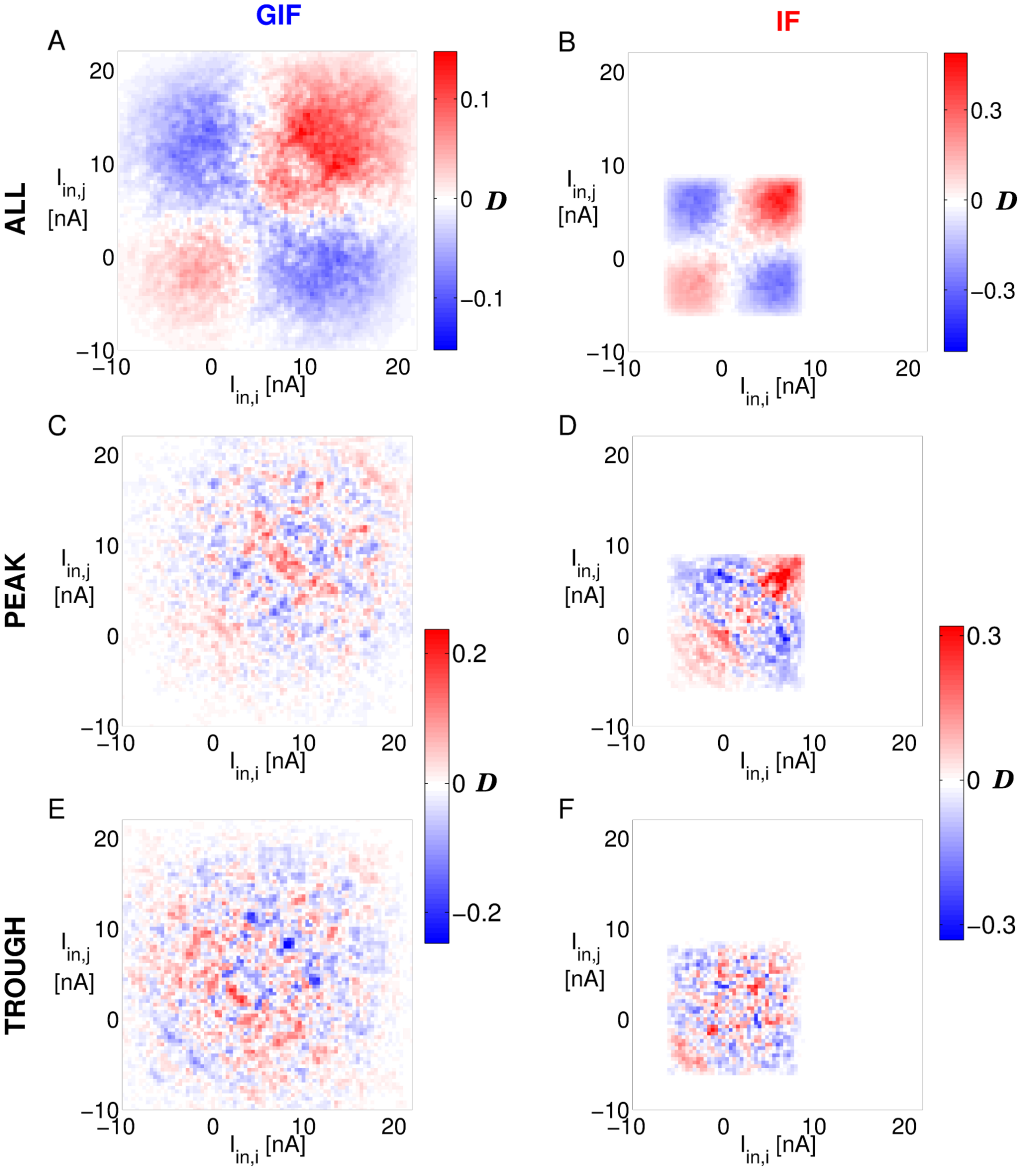
Deviation from independence of intrinsic currents for adjacent neurons. A: Deviation from independence 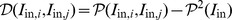 of intrinsic currents 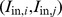 flowing through the membrane of pairs 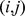 of adjacent neurons in the GIF network. Shades of red (blue) indicate 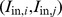 values that occur more (less) often than what expected under the assumption of independence. C, E: The same as A, but the probability density functions 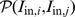 and 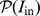 are conditioned on the peak (C) or trough (E) of the mean 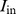 oscillation. B, D and F: The same as A, C and E, for the IF network.

Figure 7 shows that the distribution of intrinsic currents is broader and more depolarized in the GIF network, and it is more strongly modulated by the population rhythm. Furthermore, the bivariate probability density 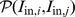 for the GIF network shows a positive deviation from independence for 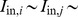 12 nA, which indicates that GIF neurons receive a strong depolarizing current which is coherent across pairs (Figure 8A). The main contribution to this depolarizing current comes from the term 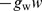. As inhibitory synaptic currents peak, individual neurons are brought to a narrow range close to the reversal potential 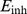, which activates the restorative variable *w* providing a post-inhibitory rebound. A positive deviation from independence is also observed in the IF network, albeit for lower (less depolarizing) values of the intrinsic currents. If probability densities are conditioned on the oscillation phase, no deviation from independence is observed, suggesting that there are no additional correlations in the intrinsic currents to cell pairs beyond those induced by the population rhythm (Figure 8C–F).

Intrinsic currents are more strongly activated in GIF neurons, and they provide a depolarizing force, coherent across neurons, that acts near the end of the inactive phase and greatly fosters oscillations. In addition to this, it is important to recognize that GIF neurons are endowed with an additional dynamical variable *w*, which actively opposes voltage changes and constitutes a single-cell memory trace of the inmediate past [38]. In IF networks, intrinsic currents only depend on the current value of the membrane potential. This results in a fixed phase relationship between the mean membrane potential and the mean intrinsic current, which are precisely in anti-phase. Conversely, the additional dynamical variable *w* in the GIF homeostatically adjusts intrinsic currents individually in each neuron on a cycle-by-cycle basis, compensating for transient variations in the input and resulting in more robust and stable oscillations.

**Figure 9:**
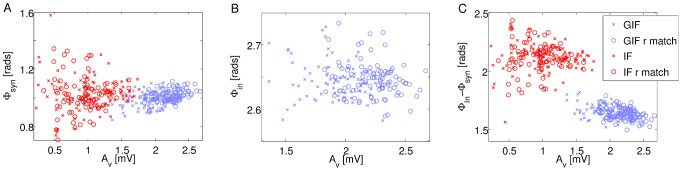
Phase relationships of synaptic and intrinsic currents and their effect on synchrony A: Phase of the sinusoidal fit to the mean synaptic current 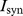 plotted against the local level of synchrony (as assessed by the amplitude of the sinusoidal fit to the mean membrane potential 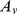). GIF: blue; IF: red. Crosses: canonical models; circles: r-matched models. B: As in A, for the mean intrinsic current 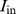. Only GIF networks are shown, as 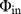 is always equal to 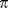 in IF networks. C: As in A, for 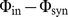.

Figure 9A shows the relative phase of the mean synaptic current with respect to the mean voltage, computed in 20 ms time windows, as a function of the amplitude of the oscillation in the mean voltage in the same window (see Methods for details). The amplitude of the oscillation in the mean voltage 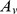 can be interpreted as a measure of oscillation strength, and it is strongly correlated to both the amplitude of the oscillation in the mean synaptic current 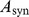 and the amplitude of the oscillation in the mean intrinsic current 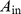. While GIF networks exhibit high oscillation strength and small variability in the phase of the mean synaptic current, IF networks exhibit lower oscillation strength and higher 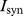 phase variability, which increases with poorer synchrony. Phase values for the synaptic current are much more variable in IF than in GIF networks, but their mean values are very similar (1 for the GIF, 1.02 for the IF). Circular-linear correlation analysis reveals that the phase of the mean synaptic current advances with higher synchrony for all networks (p<0.01), albeit less clearly in the case of the r-matched IF network (p = 0.012). Circular-linear correlation values 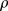 for the four networks considered are reported in Table 3, along with the corresponding p-values of the null hypothesis of no correlation.

**Table 3:** Circular-linear correlation analysis corresponding to the data plotted in Figure 9.

	IF	GIF	IF r-matched	GIF r-matched
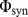 - 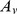	0.38 (0.0008)	0.35 (0.004)	0.32 (0.012)	0.32 (0.006)
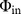 - 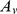	0.15 (0.33)	0.29 (0.02)	0.2 (0.16)	0.19 (0.17)
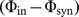 - 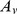	0.38 (0.0008)	0.41 (0.0006)	0.32 (0.012)	0.34 (0.004)

Circular-linear correlation has been computed between phase values of the mean synaptic current and 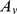 (first row, corresponding to data shown in Figure 9A), between phase values of the mean intrinsic current and 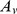 (second row, corresponding to Figure 9B), and between the phase mismatch between synaptic and intrinsic currents and 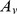 (third row, corresponding to Figure 9C). Correlation values 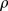 are shown for each of the four networks considered, with the p-values of the null hypothesis of no correlation shown in parenthesis.

A more striking effect of the different intrinsic neuronal properties is observed in the distribution of the relative phase of the mean intrinsic current (Figure 9B). In IF networks, intrinsic currents only depend on the current value of the membrane potential, hence their phase relationship is fixed and equal to 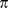. In GIF networks, conversely, intrinsic currents are adjusted on a cycle-by-cycle basis as a function of the recent input history, and peak later in the cycle, when inhibition has waned almost completely and neurons are driven by background inputs and intrinsic currents only. Depending on the background input that is received in the inactive phase of the oscillation, and in particular in its late portion, when the synaptic drive wanes, the intrinsic current will be differentially adjusted in each neuron. Since the intrinsic current in GIF neurons is restorative and tends to oppose voltage changes, its net effect will be a reduction of the variability across neurons in the membrane potential trajectories, which results in a narrower population spike, i.e. enhanced synchrony.

The phase mismatch between synaptic and intrinsic currents is a significant indicator of oscillation strength, as shown in Figure 9C. In the IF networks, the mean intrinsic current depends on the mean voltage variable only; hence, the phase mismatch between synaptic and intrinsic currents exhibits the same level of correlation with oscillation strength as the phase of synaptic current. Conversely, in the GIF networks, the phase difference 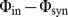 shows a stronger and more significant correlation with local synchronization (as assessed by the amplitude of the sinusoidal fit to the mean membrane potential 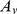) than 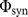, even though the phase of the mean intrinsic current 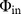 is itself independent of synchrony (Figure 9B and Table 3, second row). As 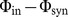 decreases, intrinsic currents peak later with respect to synaptic currents, and are more effective in bringing together the trajectories of individual neurons in the critical portion of the oscillation cycle that just precedes a population spike.

**Figure 10:**
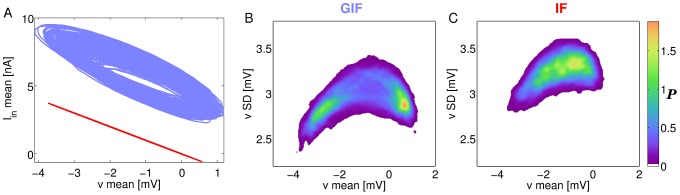
Mean and standard deviation of the membrane potential across neurons. A: Covariation of the mean membrane potential and the mean intrinsic current across neurons in the GIF (blue) and IF (red) networks. B, C: Bivariate probability density function of the mean and standard deviation of the membrane potential variable across neurons for the GIF (B) and IF (C) networks. Brighter colors indicate higher probabilities.

Figure 10 shows the covariation of the mean membrane potential and the mean intrinsic current (panel A), and the covariation of the mean membrane potential and its standard deviation (panel B, GIF; panel C, IF). In the IF neuron (red line in Figure 10A), the intrinsic current only depends on the current value of the membrane potential (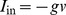). In the GIF neuron (blue line), the additional dynamical variable *w* implements a cellular memory of the inmediate past. The subthreshold dynamics of the GIF neuron is mathematically equivalent to a damped linear oscillator; hence, the trajectory in the phase plane 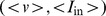 has an elliptical shape, as expected from a linear oscillator driven by noisy inputs (mediated by synaptic background inputs) with an oscillatory component (mediated by inhibitory currents originated within the network). Intrinsic currents are always greater in the GIF neuron, especially for hyperpolarized values of the membrane potential. As the inhibitory synaptic current peaks, the average membrane potential is driven close to 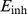, which strongly activates intrinsic inward currents. These act as a coherent depolarizing force across neurons, as the trajectory evolves clockwise in the 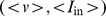 plane and the network approaches a new active phase of the oscillation.

The upward phase of the oscillation, between the trough of the membrane potential oscillation and the subsequent population spike, is a critical time window for the regulation of synchrony, as neurons are progressively released from inhibition and evolve on the basis of the background input and their intrinsic dynamics, with little influence from the local network. In this time frame, GIF neurons experience a particularly strong depolarizing drive from their intrinsic currents mediating post-inhibitory rebound, as the mean intrinsic current in the upper portion of the 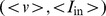 trajectory is greater than in the lower portion for equal values of the mean membrane potential. This, in turn, results in lower values for the standard deviation of the membrane potential across neurons (Figure 10, compare B and C), that is, the membrane potential trajectories of individual neurons are closer together; hence, they will cross the threshold for spike generation in a briefer time window, ultimately resulting in higher synchrony.

It is worth noting that this synchronization mechanism is different from the resonant synchronization reported for networks of coupled oscillators, where individual neurons fire regularly in each cycle. In the low-noise, mean-driven regime, the amplitude and frequency of collective oscillations strongly depend on the intrinsic frequency of individual oscillators [15]. However, if neurons are poised in the noise-driven, irregular firing regime, the intrinsic frequency of subthreshold damped oscillations have very little effect on the amplitude and frequency of collective oscillations. Rather, it is the amount of damping that most strongly affects oscillation strength, with the more underdamped subthreshold dynamics resulting in stronger oscillations (see section “Effects of variations in the intrinsic neuronal parameters and in the connection delays on synchrony” and Figure S2 in Text S1). More underdamped subthreshold dynamics imply stronger rebound from inhibition. Hence, this result further highlights the key role played by post-inhibitory rebound as the main dynamical mechanism underlying enhanced synchrony in GIF networks.

### Hyperpolarizing vs. Shunting Inhibition

In some brain regions and neuron types, especially in early stages of development, 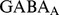 signalling has been shown to be shunting or depolarizing, rather than hyperpolarizing. That is, GABA reversal potential can be above the leak reversal potential. In particular, 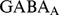 mediated inhibition has been shown to be strongly depolarizing in the developing brain [71], and remains shunting in some interneuron types of the amygdala, cerebellum, CA3 and dentate gyrus even in mature animals [47], [48], [72], [73]. Intriguingly, the polarity of GABA effects could also differ among distinct subcellular compartments [74], and be modulated on short time scales by activity-dependent mechanisms of chloride homeostasis [75], [76]. Shunting inhibition has been shown to strengthen collective oscillations in the gamma range in the presence of heterogeneity in the level of excitability across neurons [48]. However, it has been reported that neurons near a Hopf bifurcation are poorly reset by shunting inhibitory pulses [77]. In this section, we investigate how the polarity of inhibitory synaptic potentials affect the mechanisms of gamma rhythmogenesis, and whether subthreshold intrinsic oscillations are expected to enhance collective oscillations if inhibition is shunting.

**Figure 11:**
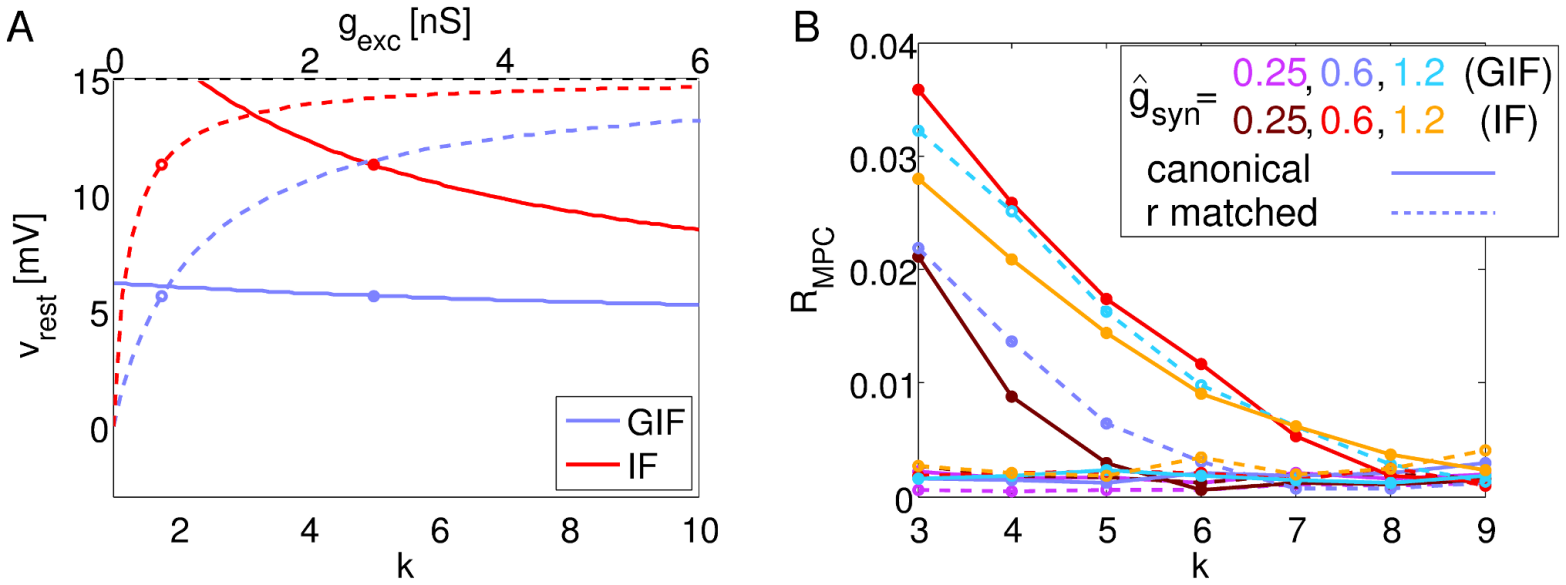
Neuronal dynamics if inhibition is shunting, rather than hyperpolarizing. A: Resting potential as a function of the background inhibition-to-excitation ratio *k* for the canonical value of 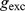 (solid lines), and as a function of the background synaptic excitation 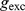 for the canonical value of 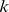 (dashed lines). Solid lines refer to the bottom axis; dashed lines to the top axis. Circles show the canonical values of the corresponding parameters. All parameters as in Table 1, except 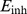 = 4 mV. Compare with analogous results for hyperpolarizing inhibition shown in Figure 1A. B: Synchrony (as assessed by 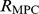) as a function of the background inhibition-to-excitation ratio 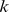 for different values of the coupling strength 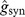. Voltage threshold for spike generation 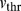 has been increased to 15 mV in the canonical models, in order to compensate for the depolarization of the resting potential and to keep the models in the fluctuation-driven regime. Voltage thresholds for r-matched models have been scaled accordingly for each value of 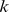. GIF: purple, weak coupling (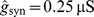); blue, medium coupling (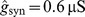); light blue, strong coupling (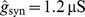). IF: brown, weak coupling; red, medium coupling; orange, strong coupling. All other parameters as in Table 1, except 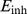 = 4 mV, 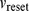 = 4 mV. Dots indicate simulated points, lines are drawn to guide the eye.

Changes in the reversal potential of synaptic conductances do not affect neuronal eigenvalues, since the systems (2) and (3) are linear, but do affect the resting potential in response to constant background input (see equation (A-1) in the Appendix, Text S1), and hence the distribution of the membrane potential in response to noisy synaptic bombardment. In particular, the resting potential in response to constant background conductances is considerably more depolarized in the IF neuron, as expected from the restorative character of the resonant variable in the GIF neuron (Figure 11A, compare with the analogous results for hyperpolarizing inhibition shown in Figure 1A).

As expected from the theoretical considerations and numerical simulations presented above, this difference in membrane potential distribution responses confers a synchronization advantage to the IF neuron, which indeed exhibits stronger synchronization in a broad range of parameter space if inhibition is shunting (Figure 11B). In fact, for the canonical coupling value 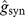 = 0.25 mV, only IF networks exhibit a noticeable level of synchronization (brown solid curve), and only for low values of 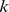, which corresponds to greater depolarization and consequently higher firing rates. Stronger coupling results in higher synchrony in canonical IF networks, which saturates and eventually slightly decreases, as previously observed in the case of hyperpolarizing inhibition (Figure 5B). Conversely, GIF networks exhibit appreciable oscillations only for medium to high values of the coupling strength, and only if the voltage threshold 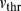 has been adjusted to increase their firing rate responses to background inputs to the IF level (r-matched GIF, blue and light blue dashed lines). Even in these conditions, the level of synchrony observed in IF networks is only reached for high values of the coupling strength.

This effect is reminiscent of the phenomenon reported by Börgers *et al.* [77], who showed that neurons with subthreshold oscillations are poorly reset by shunting inhibitory pulses. The main difference between their approach and ours is that they considered a mean-driven, tonic spiking regime, while we considered a fluctuation-driven regime. In their model, as in ours, volleys of inhibition bring neurons close to the reversal potential of inhibition, which has a synchronizing effect. However, in their model, as inhibition wanes the fixed point (which is a focus for a neuron with subthreshold damped oscillations) becomes weakly repelling. As the focus undergoes a bifurcation from weakly attracting to weakly repelling, a “ghost” attractor dominates the dynamics in its vicinity. Hence, small differences in initial conditions between different neurons are amplified, as different neurons might make a different number of turns around the weakly repelling focus before leaving its vicinity and start a new spiking trajectory. This effect is more pronounced in the absence of external noisy inputs, since strong background conductances would move the state variables away from the bifurcating focus, into regions of phase-space with stronger and more directive field.

In our model, a slightly depolarized reversal potential for inhibition abolishes post-inhibitory rebound excitation, and actually results in post-excitatory rebound inhibition for those cells with 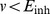. This situation is observed even if the resting potential (defined as in section “Single neuron dynamics” as the membrane potential 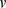 that satisfies 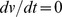 in systems (2) and (3) with constant background input) is above 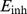, due to the fluctuating nature of background conductances. The hyperpolarizing current resulting from post-excitatory rebound inhibition pushes neurons away from the spiking threshold and enhances the desynchronizing effect of the noisy background input, by lengthening the time window during which cells evolve free from inhibition, driven solely by the incoherent background input. This phenomenon gives GIF networks a synchronization disadvantage with respect to IF networks, in addition to the synchronization disadvantage resulting from smaller firing rate and membrane potential distribution responses to the noisy background input, the latter effects being due to the presence of a restorative current.

## Discussion

Oscillations in the gamma range (30–100 Hz and higher) have been the focus of intense experimental and theoretical work for more than two decades (reviewed in [1]–[4]). Synchronization in that frequency range has been proposed as a physiological substrate of perceptual binding, whereby individual neurons selective to different features that coactivate in the same gamma cycle would signal the coherent perception of those features, i.e., when those features belong to an object that is perceived as a single entity [78]. Gamma band oscillations are not exclusive to sensory cortices, but have also been observed in high-level decision areas such as the medial prefrontal cortex, in areas related to working memory maintainance such as the lateral intraparietal area, and in non-cortical regions such as the hippocampus, some subcortical nuclei, and the spinal cord. More recently, gamma-band synchronization has been recognized as a general process of neuronal processing, which might enable selective, dynamic and flexible routing of information across brain regions [79], [80]. In accordance with its putative role in cognition, alterations of neuronal coherence in the gamma band have been associated with several psychiatric disorders, including autism and schizophrenia (see [81] for a recent review).

In spite of the recognized key role of high-frequency oscillations in neuronal processing, the biophysical mechanisms that underlie their generation are still incompletely understood. In particular, the role of intrinsic subthreshold oscillations, which have been observed in several interneuronal types critically involved in the emergence of gamma oscillations, is still unclear.

Here, we show that intrinsic subthreshold oscillations enhance the synchrony induced by hyperpolarizing inhibitory coupling in networks of irregularly firing interneurons. As inhibitory synaptic currents peak, neurons are brought together to a narrow range close to the reversal potential 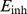. If neurons are endowed with damped subthreshold oscillations, hyperpolarization activates inward currents and results in post-inhibitory rebound, which in turn induces a depolarization of the membrane potential that is coherent across neurons due to common inhibitory input. This translates to a higher synchrony of spiking activity.

Intrinsic subthreshold oscillations can result from delayed restorative currents, and are enhanced by the additional presence of amplifying currents [29]. Llinás *et al.* described a mechanism based on the interplay between a persistent sodium conductance and a delayed-rectifier potassium conductance [27]. Another current that often results in oscillatory properties is the *h* current, a hyperpolarization-activated inward current which has been proposed to control rythmogenesis in neurons and cardiac cells [82]–[85], and is also expressed in fast-spiking interneurons of the hippocampus [86]. Activation of 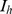 in response to IPSPs might induce the post-inhibitory rebound that is the key mechanism underlying enhanced synchrony in GIF networks. Deinactivation of the low-threshold inward calcium current 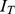 might play a similar role. In fact, several biophysical mechanisms can yield equivalent neuronal dynamics [87]. The adoption of phenomenological models like the IF and GIF neurons enables us to assess the role of subthreshold damped oscillations in a general framework, abstracting from the specific biophysical mechanisms that are responsible for their generation.

The heterogeneity of neuronal types is a phenomenon observed in many brain regions, and especially in those that are phylogenetically more recent and thus posited to be involved with higher brain functions, such as the hippocampus and the neocortex [24], [25]. The functional significance of neuronal heterogeneity is an important, yet barely explored question that can greatly benefit from theoretical and computational approaches. As a step toward understanding the functional relevance of the complex distribution of intrinsic neuronal properties observed in the brain, we need to develop a better understanding of the effects of intrinsic neuronal properties in collective network dynamics in simplified settings. In general, the modification of a specific intrinsic neuronal property (e.g., modifying the subthreshold dynamics from purely passive to exhibiting damped oscillations) results in changes in several other intrinsic properties (e.g., firing rate and depolarization responses to noisy synaptic bombardment). The latter changes can have substantial effects on the resultant network dynamics, which could be of the same or greater magnitude than the effects of the specific property under investigation. Hence, it is crucial to develop methods that enable a *selective* modification of a specific neuronal property, in the absence of changes in other neuronal properties that could also have a significant effect on the resultant network dynamics. This aim motivates our modelling choice of using IF and GIF neurons, because these models enable precise tuning of the firing rate response to noisy inputs by changes in the voltage threshold for spike generation, without affecting the subthreshold dynamics. In principle, the same aim could also be accomplished using more complex and realistic models, appropriately chosen from a large population generated with a database approach [88]. However, the highly non-linear dependency of neuronal activity on model parameters and initial conditions, which generally increases with model complexity, will have to be taken into account [89].

We identified three factors, conceptually independent but related through subthreshold intrinsic dynamics, that affect the influence of single-neuron properties on synchronization mediated by inhibition: *i*) the firing rate response, *ii*) the membrane potential distribution, in particular its relationship with the reversal potential of inhibition, and *iii*) the shape of IPSPs, in particular the presence of a sign inversion (post-inhibitory rebound depolarization or post-excitatory rebound inhibition). Importantly, the adoption of phenomenological models with a fixed voltage threshold for spike generation enabled us to disentangle the contribution to synchronization of these different factors.

We presented some illustrative examples that expose each of these factors separately. By adjusting the firing threshold in order to keep the firing rate response equal for different values of the membrane potential distribution, we could isolate the influence of the membrane potential distribution on synchronization, and show that a more depolarized membrane potential distribution results in higher synchrony because of a stronger electrochemical driving force, independently of firing rate (Figure 5B and C, compare curves corresponding to the same neuron type and different background inhibition-to-excitation ratio *k*).

By comparing the synchronization properties of networks with different inhibition-to-excitation ratios *k*, with or without the additional calibration of the voltage threshold 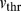 in order to match the firing rate response, we showed that higher firing rates increase synchronization regardless of the membrane potential distribution (Figure 5B and C, compare solid and dash lines of the same color. Dash lines correspond to higher firing rate response to the noisy background for the GIF neuron, while the opposite holds for the IF neuron). Higher firing rates result in stronger inhibitory currents in each cycle of the oscillation which more effectively reset the membrane potential to a narrow range near the inhibitory reversal potential 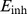.

By comparing the synchronization properties of networks of GIF and IF neurons, with or without the additional calibration of 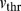 in order to match the firing rate response to the noisy background, we exposed the additional synchronizing effect due to the IPSP shape, in particular to the post-inhibitory rebound associated with hyperpolarizing IPSP in the GIF neuron (Figure 5B and C, compare curves corresponding to the same firing rate response to the noisy background and inhibition-to-excitation ratio 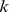, and different subthreshold dynamics, such as the solid blue line for the GIF and the dash red line for the IF). If the reversal potential is slightly above the leak reversal potential (shunting inhibition), IPSPs are slightly depolarizing. In this scenario, the presence of intrinsic subthreshold oscillations in the GIF neuron results in IPSP-mediated post-excitatory rebound inhibition, effectively diminishing the strength of oscillations in GIF networks (Figure 11B).

While subthreshold damped oscillations and post-inhibitory rebound always coexist in the GIF neuron, due to the linear description of the subthreshold dynamics, real neurons and non-linear neuron models can display post-inhibitory rebound while still responding passively to weak inputs. For example, a neuron can display real eigenvalues in the linearization of its subthreshold dynamics around its resting potential, while still being endowed with a hyperpolarization-activated inward current that only activates at membrane potentials considerably lower than its resting potential. In this case, weak hyperpolarizing inputs will elicit purely passive responses, while strong inputs will elicit post-inhibitory rebound and the neuron will return to baseline with a trajectory that overshoots its resting potential. Our results suggest that this class of neurons will also display enhanced propensity towards collective oscillations when coupled by hyperpolarizing inhibition. In this case, we would predict a non-linear increase in synchrony as a function of coupling strength, with a boost in synchrony as neurons switch from linear, passive responses to hyperpolarization to non-linear responses mediated by post-inhibitory rebound.

The adoption of a network model with spatial extension enables one to assess the distance-dependent modulation of synchrony (Figure 4). Distance-dependent delays do not introduce a strong bias in the synchronization properties of cell pairs across short distances (up to ∼1 mm), as higher input correlations to adjacent neuron pairs are counterbalanced by the desynchronizing effect of short-latency inhibition, resulting in a flat profile at the level of membrane potential correlation. As a result, firing synchrony only shows a modest decrease at short distances, but is otherwise distance-independent. Hence, the spatial profile of network synchronization does not exhibit a consistent topological structure, unless such a structure is present in the input. This is a desirable property, since spurious correlations would disrupt an efficient representation of information. The mechanism we describe is different from the recently proposed decorrelation by cofluctuations in excitation and inhibition [69], [90], and is crucially dependent on the spatial dimension of the network, in particular on distance-dependent propagation delays.

As background input to individual neurons, we consider noisy conductances without any spatial correlation, and with only rapid temporal correlations consistent with filtering by fast AMPA and 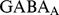 synapses. Spatio-temporal correlations in the input, either induced by the statistics of sensory stimuli or generated by neuronal dynamics in other brain areas, are expected to affect our results significantly. In fact, input temporal correlations affect neuronal processing in a cell-specific way [91], [92] and spatial correlations shape the activity of recurrent networks [93]. Hence, we predict that the inclusion of more complex and realistic patterns of spatio-temporal correlations in the background input will enhance the cell-type dependent effects reported here.

The study of how intrinsic neuronal properties affect the dynamics of networks receiving spatio-temporally structured inputs is an important topic for future research. In particular, some areas of the brain that are known to form allocentric maps of space, such as the hippocampus and entorhinal cortex, display a broad variety of interneuronal types. In some cases, intrinsic neuronal properties correlate with neurometric features of the maps, as in the case of the correlation between 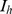 time constant, intrinsic oscillation frequency and grid field spacing in the dorsal region of the entorhinal cortex [94]–[96]. An extension of the current approach that includes spatio-temporally structured inputs and synaptic plasticity rules could be highly valuable to our understanding of how intrinsic properties and plasticity processes interact with the statistics of external inputs in the formation, access and manipulation of maps in the brain.

In this work, we consider inhibitory networks with all-to-all connectivity and equal weights. This choice is convenient to assess the effects of propagation delays in the absence of additional spatial structure, which might dominate the dynamics in more realistic conditions. Our aim is to characterize the dynamical constraints enforced by delayed inhibitory coupling, while keeping all other parameters as unspecific as possible. Even if inhibitory connectivity in the neocortex might be very dense, almost approaching the all-to-all connectivity considered here (see [97], [98] and references therein), the heterogeneity of synaptic weights is expected to be significant. In our model, higher input correlations for nearby neurons interact with short latency inhibition, resulting in a flat spatial profile of output correlations. In a more realistic scenario, we expect that neurons that share a higher number of presynaptic partners will be more likely to synchronize [99], especially if they are not connected or if they are located at a distance greater than required for short latency mutual inhibition. It should be noted that heterogeneous connectivity would break the toroidal symmetry of the network, and would greatly complicate an exhaustive characterization of the resultant dynamics.

Likewise, interactions between excitatory and inhibitory neurons can also have a great impact on the population activity. If the connections from the excitatory to the inhibitory population are weak, or if excitatory neurons fire at low rates, the network would still operate in the ING (Interneuron Network Gamma) regime, and we would expect modest deviations from the results reported here. However, in the case of PING (Pyramidal - Interneuron Network Gamma) oscillations, excitatory neurons are active and they project to the inhibitory population. The dynamic interplay between excitatory and inhibitory populations could give rise to a qualitatively different collective dynamics, which could diverge substantially from the purely interneuronal network dynamics we described. The closed-loop interaction between excitatory and inhibitory populations greatly complicates the dynamics and the mechanistic analysis of the effects of subthreshold intrinsic oscillations in either, or both, neuronal populations, in the emergent collective rhythm. While the current study builds a useful foundation for pursing these investigations, the analysis of this case is beyond the scope of this manuscript and will be presented in a separate article.

It is worth highlighting that the ING mechanism is not only of theoretical interest, but has also been supported by experimental data (see, for example, [6]). In fact, the extent to which excitatory neurons contribute to the establishment and regulation of high-frequency oscillations is still a matter of debate [100]. Conversely, the necessary role of inhibition has long been established [6], [7], [68]. The role of inhibitory interneurons in the generation and modulation of high-frequency oscillations deserves special attention, since several neuropsychiatric disorders are associated with disruption of gamma band coherence and corresponding alterations in interneuron properties [101]. In fact, the key role played by interneuronal dysfunctions in the etiology of several neurological and psychiatric diseases has led to the introduction of the word “interneuronopathies”, which hints to underlying commonalities in genes and developmental mechanisms specific to GABA-ergic signalling (in particular, those related to the fine tuning of excitatory/inhibitory balance along the course of development) shared by several disorders with vastly different phenomenology, such as autism, epilepsy and schizophrenia [102]–[106].

In this work, we have focused on the influence of intrinsic subthreshold oscillations in the generation of high-frequency oscillations in interneuronal networks. In our approach, the desynchronizing effect is provided by the incoherent background input. Post-inhibitory rebound enhances synchrony by providing a depolarizing current which is coherent across cells due to common inhibitory input (Figure 10A), and hence can counteract the desynchronizing effect of the incoherent background input. Other authors have identified several other factors that can either impair or promote high-frequency oscillations. Neuronal heterogeneity, either due to heterogeneity in the excitability of individual neurons, in their connections, or due to small network size or local coupling, is well known to have a desynchronizing effect [11]–[13], [17], [107]. Conversely, gap-junctional coupling among interneurons [108] and shunting inhibition [48] have been shown to increase synchrony by homogenizing firing rates across neurons with heterogeneous excitatory drive poised in the regular firing regime. In particular, gap-junctional coupling seems to bear a close resemblance to the synchronization mechanisms we described here, since both post-inhibitory rebound and gap-junctions can evoke depolarizing currents in the target cell, in the absence of excitatory chemical synaptic connections. However, the two mechanisms are markedly different. While gap-junctions tend to diminish the distance between the membrane potential trajectories of coupled neurons regardless of spiking activity, post-inhibitory rebound is spike-mediated. The investigation of how these different factors interact in realistic neuronal networks is an important topic for future research.

Fast spiking basket interneurons exhibit several dynamical properties that have been suggested to facilitate gamma oscillations. For example, they can sustain high-frequency spiking with little or no adaptation [109], display fast synaptic kinetics of both incoming and outgoing synapses [19], [26], [110], and are endowed with specific intrinsic properties that boost the transmission of fast and synchronous EPSPs through their dendrites [111]. Fast spiking interneurons have also been shown to exhibit membrane resonance [30], type II *fI* curves [34], and type II Phase Response Curves [112]. Our modeling effort does not aim to reproduce all the dynamical features that have been reported in these cells. Rather, our aim is to elucidate the influence of a specific and commonly observed intrinsic neuronal characteristic, subthreshold damped oscillations, in the emergence and properties of high-frequency oscillations. In accordance with this intention, we adopted the simplest phenomenological model that can capture this dynamical property. Importantly, the IF and GIF models we considered in this study differ in their subthreshold dynamics (passive in the IF, with subthreshold damped oscillations in the GIF), but they both exhibit type I Phase Response Curves.

Recently, Moca *et al.* studied the effect of interneuronal membrane resonance in the gamma frequency synchronization of networks of excitatory and inhibitory neurons [16], and reported more stable oscillations in networks with resonant interneurons, in general agreement with our results. However, our approach differs in two fundamental aspects. In our model, individual neurons are driven by strong barrages of background excitatory and inhibitory noisy conductances, mimicking neuronal activation *in vivo*; hence, neurons are poised in the irregular firing regime. As we have shown, the interaction between intrinsic neuronal properties and background synaptic conductances is a key factor in the resulting network activity. Conversely, Moca *et al.* included only a modest level of noise in their simulations, whose main effect is the generation of variability across trials. More importantly, their study considered collective oscillations generated in the regular firing regime, in which every neuron takes part in every cycle of the population rhythm (see, for example, Figure 4B in [16]). If individual neurons are poised in the regular firing regime, the synchronization properties of the network will depend on the geometry of the limit cycle or chaotic attractor corresponding to tonic spiking, rather than on subthreshold dynamics themselves. Membrane resonance often results in a bifurcation to tonic spiking where firing period depends only weakly on input parameters, such as an Andronov-Hopf bifurcation. However, resonant subthreshold dynamics do not always correspond to a tonic spiking attractor with stable periodicity. For example, a neuron model characterized by a saddle node bifurcation off invariant circle will exhibit stable firing frequency in the tonic spiking regime, but passive subthreshold dynamics [87]. The correspondence between subthreshold dynamics and tonic spiking activity is expected to be even less accurate as more realistic neuronal models, and real living cells, are considered [112]. In particular, the neuron models we considered in this study behave similarly in the regular firing regime (see section “Phase Response Curves in the IF and GIF neuron” and Figure S1 in Text S1). Correspondingly, their synchronization properties do not differ consistently if neurons fire regularly in each cycle (not shown).

During network oscillations, pyramidal cells fire sparsely, while interneurons are thought to emit action potentials in every cycle. However, most experimental evidence on interneuron dense firing comes from *in vitro* studies where strong oscillations are induced by application of a glutamatergic agonist [6] or manipulation of the ionic environment [113]. Furthermore, most of these studies employed extracellular recordings, which are biased towards neurons with strong firing activity. In fact, an experimental study in rats engaged in running and exploration reported selective and sparse firing also in interneurons ([114], see in particular their Figure 1C). Other studies in rats reported sparse interneuronal firing during sensory-evoked gamma responses in the olfactory bulb *in vitro* [115], and very sparse interneuronal firing during isoflurane anesthesia [116]. Intracellular recordings from interneurons in hippocampal slices activated by the cholinergic agonist carbachol also reported single-cell firing rates that are two or three times lower than collective gamma frequency [117]. We believe that this regime of partial synchronization might be, at least, as relevant to natural neuronal computation as the strongly synchronous bouts of gamma activity observed in response to the presentation of “favorite” stimuli in early sensory cortexes [5]. In fact, the level of synchrony can be modulated by physical properties of the stimulus, such as contrast [118]. Furthermore, weakly synchronous states are both information-rich (in terms of the output they can convey to other brain regions) as well as information-sensitive (in terms of the representation capabilities they offer when stimulated by temporally structured inputs [119]). Hence, while the presentation of optimal stimuli in laboratory settings might induce strong gamma oscillations, neuronal information processing in naturalistic conditions might operate in an intermediate regime of information-rich weakly synchronized oscillations.

Certain neuromodulators can affect the intrinsic properties of neurons. In particular, acetylcholine (ACh) changes the PRCs of cortical neurons by down-regulating the M-current, a slow potassium current which is also related to subthreshold oscillations [120]. Since subthreshold oscillations enhance oscillation strength in networks of interneurons coupled by hyperpolarizing, but not shunting, inhibition, our results suggest the intriguing possibility that ACh could differentially regulate the level of synchrony in different brain regions, depending on the nature of local coupling. For example, GABAergic input onto interneurons is shunting in the amygdala, CA3 and dentate gyrus [47], [48], [72], but can be either shunting or hyperpolarizing in the cerebellum [73], and is hyperpolarizing in the neocortex [97]. These region-specific effects could induce a bias in the synchronization properties of local networks and hence in the effective coupling between brain regions, under neuromodulatory control [80]. Experimental efforts in this direction would greatly benefit from theoretical investigations aiming to elucidate the properties and communication mechanisms of interacting networks [119], [121].

Experimental data in the hippocampus and other areas revealed that distinct interneuronal populations are active at different phases of ongoing network oscillations, innervate specific postsynaptic types and subcellular domains, and might contribute to different aspects of information processing [24], [116], [122]. Reductionist modelling approaches combined with optogenetic experimental techniques will be needed in order to gain a mechanicistic understanding of the complex interaction between single-cell morphology, physiology and the emerging function of neuronal microcircuits.

## Supporting Information

Text S1 This file includes sections “Phase Response Curves in the IF and GIF neuron”, “Effects of variations in the intrinsic neuronal parameters and in the connection delays on synchrony”, and “Appendix”; and Figures S1 and S2.
(PDF)
