# Supplementary material for: Interplay of Intrinsic and Synaptic Conductances in the Generation of High-Frequency Oscillations in Interneuronal Networks with Irregular Spiking
Source: PLoS Comput Biol. 2014 May 1;10(5):e1003574. doi: 10.1371/journal.pcbi.1003574 (PMC4006709; doi:10.1371/journal.pcbi.1003574)
Supplement: Text S1 — This file includes sections “Phase Response Curves in the IF and GIF neuron”, “Effects of variations in the intrinsic neuronal parameters and in the connection delays on synchrony”, and “Appendix”; and Figures S1 and S2. (PDF) [file pcbi.1003574.s001.pdf]

Phase Response Curves in the IF and GIF neuron

Many neurons and models can be made to fire repetitively and regularly with the injection of a constant depolarizing current. A small perturbation, either excitatory or inhibitory, alters the duration of the current cycle by an amount that depends on the phase at which it is delivered. This concept lies at the core of Phase Response Curve (PRC) theory, which has yielded important insights into the synchronization properties of coupled oscillators (recently reviewed in [44]). Neurons are said to exhibit a type I PRC if an excitatory perturbation will always advance their current phase. Conversely, neurons with a type II PRC are delayed by a depolarizing pulse received early in the cycle. The latter neuron type has been shown to synchronize more easily when coupled by excitation. This phenomenon can be easily understood intuitively by considering a pool of weakly connected neurons oscillating roughly synchronously. In any given network cycle, neurons that fire before most of their peers will receive most PSPs in the early portion of their individual cycle, and will be delayed. Conversely, neurons that fire after most of their peers will receive most PSPs in the late portion of their cycle, and will be advanced. Overall, the population will be more tightly synchronized in the next cycle. By the same argument, a type II PRC is expected to have a desynchronizing effect when neurons are coupled by inhibition. In this scenario, type I PRC neurons, which exhibit phase shifts in the same direction for all phases, are expected to synchronize more robustly.

We set the background noisy conductances to zero and injected a constant depolarizing current $\mathrm{I_{bias}}$=27.6 nA to the right-hand-side of equation (2) (for the IF) and to the equation for v in the system (3) (for the GIF), in order to elicit regular tonic spiking at 118 Hz. While the GIF model has a voltage threshold for spike generation equal to 6.3 mV, as in the canonical model used throughout the main document, we set v_{thr} to 13.4 mV in the IF in order to yield the same firing rate as in the GIF model. Then, we applied inhibitory exponentially decaying conductances of maximal amplitude $\hat{g}_{\mathrm{inh}}$=0.2 nS and time constant τ_{inh}=1 ms at different phases of the repetitive spiking oscillations, and measured the resulting phase change as ($\mathrm{T_{pert}}$-T)/T, where T is the unperturbed oscillation period, and $\mathrm{T_{pert}}$ is the duration of the perturbed cycle. Both the IF and GIF neurons considered in this study exhibit type I PRC, where phase delays occur in response to hyperpolarizing pulses at all phases (Figure S1). As expected from a lack of difference in their PRC type, the IF and GIF neurons do not exhibit consistently different synchronization properties when coupled by mutual inhibition, if neurons are poised in the regular firing regime (not shown).

Effects of variations in the intrinsic neuronal parameters and in the connection delays on synchrony

In the theory of coupled oscillators, the precise value of the intrinsic frequency of individual oscillators is critical for the network dynamics. However, the neuron models we considered in this study do not behave like self-sustained oscillators. While the IF neuron exhibits purely passive subthreshold dynamics, the GIF neuron exhibits subthreshold damped oscillations. The mechanisms by which GIF neurons synchronize more than IF neurons when coupled by hyperpolarizing inhibition are mainly due to the presence of post-inhibitory rebound, as explained in detail in the main manuscript, rather than to a resonant interaction between the single-cell and the network frequencies.

In this section we varied the propagation delays, which are the main determinants of the collective oscillation frequency, along with the intrinsic properties of the model neurons. The models we considered in this study are dynamically redundant, such that the same dynamical modification can be achieved by several combinations of parameter changes. Hence, we show our results in the space of model eigenvalues, where μ corresponds to the real part of the eigenvalues (with opposite sign), and ω corresponds to the imaginary part. While μ represents the membrane rate constant, ω is the intrinsic oscillation frequency. The only intrinsic parameters that have been varied are g and g_{w}. Delays have been multiplied by a factor k_{d} equal to 0.8 (shorter delays, faster network oscillations), 1 (canonical value) or 1.2 (longer delays,

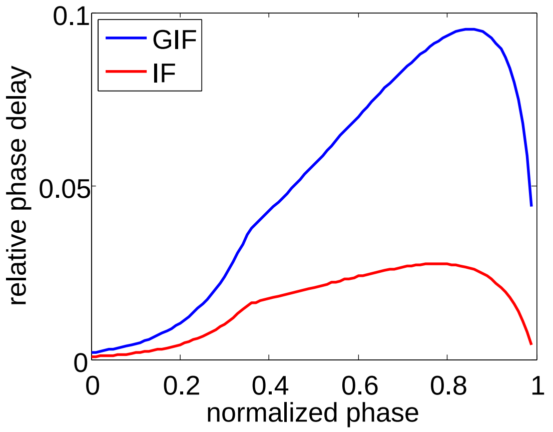

Figure S1. PRCs of the GIF and IF in response to inhibitory exponentially decaying conductances of maximal amplitude $\hat{g}_{\mathrm{inh}}$=0.2 μS and time constant τ_{inh}=1 ms. PRCs are displayed as percentual change in cycle period between perturbed and unperturbed cycles, with positive values indicating phase delays. Both models exhibit type I PRC (inhibitory perturbations always result in a phase delay).

slower network oscillations). Both the distance-dependent and the distance-independent components of the delays have been multiplied by the same factor.

Figure S2 shows the network synchrony (as assessed by R_{MPC}), the single-cell average firing rate r_{s} and the collective oscillation frequency r_{n} as a function of the intrinsic frequency ω in the GIF model with canonical membrane rate constant (μ=100 Hz, solid lines, light blue, blue and purple). We also consider an additional GIF model where the membrane rate constant has been decreased by a factor of 4, hence resulting in more weakly damped oscillations (μ=25 Hz, solid lines, light green, green and dark green). We observe a marked increase in synchrony as ω is increased, with the GIF model with slow membrane rate constant exhibiting higher synchrony for all values of ω.

Note, however, that as we increase the oscillation frequency ω, the damping coefficient $C_{\mathrm{damp}} = \mathrm{e}^{-2\pi\frac{\mu}{\omega}}$ (defined as the ratio between the second and the first peak in the free evolution of the voltage variable from an initial condition different than rest) also increases, and consequently the oscillating character of the neuron (see, for example, [38]). In order to separate the effects due to μ, ω and to the damping coefficient C_{damp}, we also consider an additional set of models where ω and μ have been covaried in order to keep a constant μ/ω ratio of 0.5, as in the canonical GIF model (dash lines, light blue, blue and purple).

The comparison between the level of synchrony obtained in the GIF models with canonical membrane rate constant (solid lines, light blue, blue and purple), with the more underdamped membrane rate constant (solid lines, light green, green and dark green), and with canonical damping coefficient (dash lines, light blue, blue and purple) reveals that it is the amount of damping that most strongly affects oscillation strength, with the most underdamped subthreshold dynamics resulting in stronger oscillations. Decreasing the membrane rate constant also increases oscillations in the IF model. In fact, in the limit of an extremely fast membrane rate constant, the IF model would follow the noisy background input instantaneously, preventing the emergence of coherent collective oscillations. However, this effect is small if compared with the effect of the damping coefficient C_{damp} in the GIF model.

It is worth noting that no sign of a resonant interaction between intrinsic and network frequencies is observed as ω is varied. The model with fixed μ/ω ratio exhibits a non-monotonous, but weak, dependence on ω. However, the value of ω that results in highest synchrony increases for longer delays. Hence we observe no decrease, but rather a slight increase, in the optimal intrinsic frequency as network frequency is

decreased, which is not consistent with a resonant interaction between intrinsic and network frequencies.

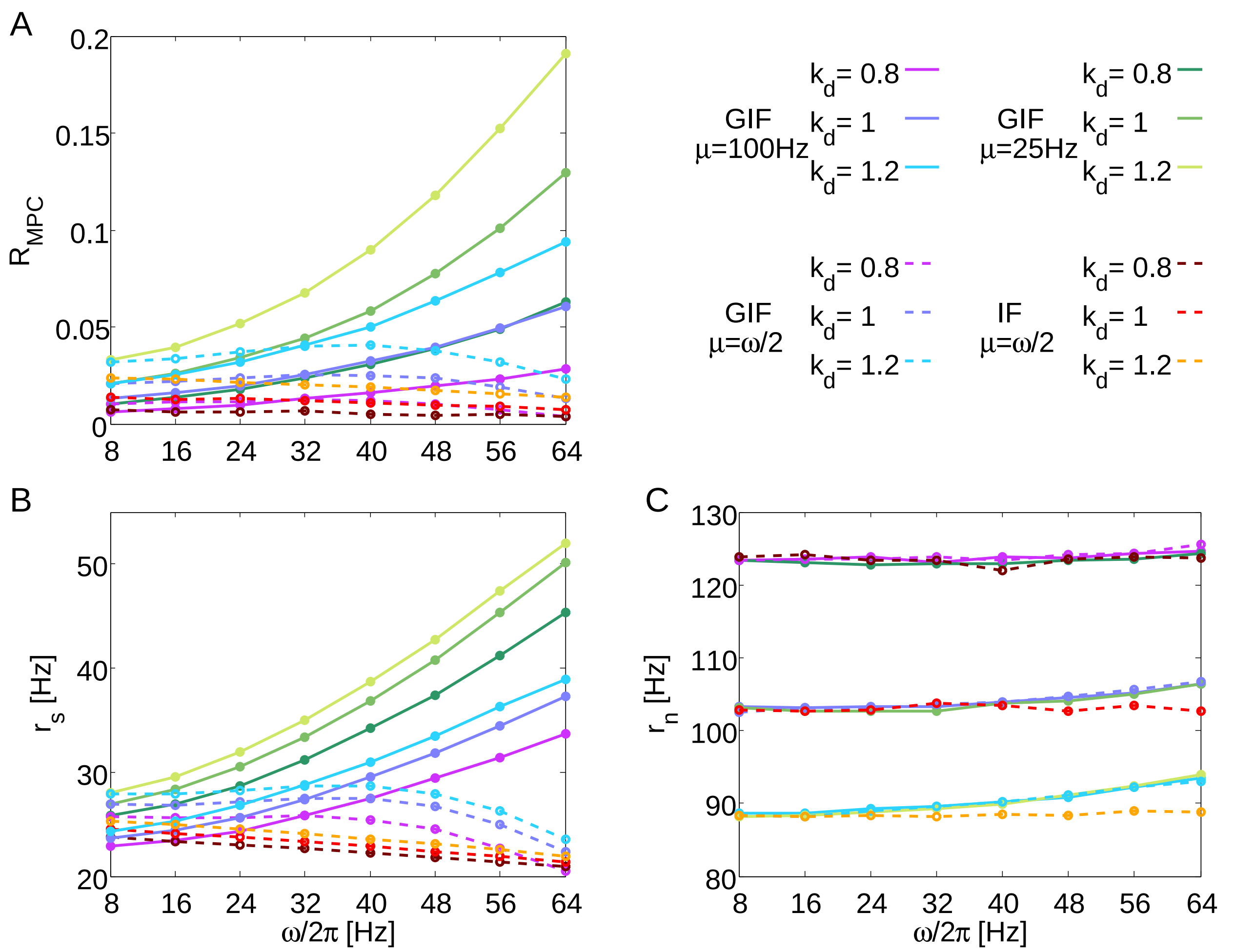

Figure S2. Effects of intrinsic neuronal parameters and connection delays on network dynamics. Synchrony (as assessed by R_{MPC}, A), firing rates (B) and network frequency (C) as a function of the intrinsic frequency $\omega/2\pi$, for three values of the connection delays and four sets of neuron models. Color and line style code as indicated. Dots indicate simulated points, lines are drawn to guide the eye.

Appendix

In this section we report the analytic expressions for the resting potential and the system eigenvalues of the model neurons with fixed background conductances (σ_{inh}=σ_{exc}=0), graphically represented in Figure 1 of the main text. Expressions are derived for the GIF neuron only, as the IF neuron can be considered a special case of the GIF neuron with g_{w}=0.

Resting potential of the GIF neuron in the presence of constant background conductances

The resting potential v_{rest} is the voltage value for which the time derivatives in system (3) are identically zero. Setting the *rhs* in system (3) to zero and solving for v yields:

$$v_{\mathrm{rest}} = \frac{g_{\mathrm{exc}} E_{\mathrm{exc}} + g_{\mathrm{inh}} E_{\mathrm{inh}}}{g + g_{\mathrm{w}} + g_{\mathrm{exc}} + g_{\mathrm{inh}}}. \tag{A-1}$$

Eigenvalues of the GIF neuron in the presence of constant background conductances

Following standard linear algebra techniques, we calculate the eigenvalues of the system (3) as the complex numbers $\lambda_{1,2}$ that satisfy

$$\det(\mathbf{J}_f - \lambda \mathbf{I}) = \mathbf{0},$$

where $\mathbf{J}_f$ is the Jacobian of the system (3), $\mathbf{I}$ is the identity matrix, and $\det(\cdot)$ is the determinant operator. Solving for λ yields:

$$\lambda_{1,2} = -\frac{1}{2}\left(\frac{g_t}{C} + \frac{1}{\tau_{\mathrm{w}}}\right) \pm \frac{1}{2C\tau_{\mathrm{w}}} i \sqrt{4 g_{\mathrm{w}} \tau_{\mathrm{w}} C - (g_t \tau_{\mathrm{w}} - C)^2}, \tag{A-2}$$

where i is the imaginary unit and $g_t = g + g_{\mathrm{exc}} + g_{\mathrm{inh}}$. The effective membrane time constant τ_{eff} and the effective intrinsic frequency f_{eff} are defined from the eigenvalues $\lambda_{1,2}$ as:

$$\tau_{\mathrm{eff}} = -1/\Re(\lambda_1) = \begin{cases} \frac{2C\tau_{\mathrm{w}}}{g_t \tau_{\mathrm{w}} + C} & \text{if } 4 g_{\mathrm{w}} \tau_{\mathrm{w}} C \geq (g_t \tau_{\mathrm{w}} - C)^2, \\ \frac{2C\tau_{\mathrm{w}}}{g_t \tau_{\mathrm{w}} + C + \sqrt{(g_t \tau_{\mathrm{w}} - C)^2 - 4 g_{\mathrm{w}} \tau_{\mathrm{w}} C}} & \text{otherwise.} \end{cases} \tag{A-3}$$

$$f_{\mathrm{eff}} = \Im(\lambda_1)/2\pi = \begin{cases} \frac{1}{4\pi C \tau_{\mathrm{w}}} \sqrt{4 g_{\mathrm{w}} \tau_{\mathrm{w}} C - (g_t \tau_{\mathrm{w}} - C)^2} & \text{if } 4 g_{\mathrm{w}} \tau_{\mathrm{w}} C \geq (g_t \tau_{\mathrm{w}} - C)^2, \\ 0 & \text{otherwise.} \end{cases} \tag{A-4}$$

where λ_1 is the eigenvalue with the greater real part in absolute value.
